# Astrocyte-to-neuron H_2_O_2_ signalling supports long-term memory formation in *Drosophila* and is impaired in an Alzheimer’s disease model

**DOI:** 10.1038/s42255-024-01189-3

**Published:** 2025-01-24

**Authors:** Yasmine Rabah, Jean-Paul Berwick, Nisrine Sagar, Laure Pasquer, Pierre-Yves Plaçais, Thomas Preat

**Affiliations:** Energy & Memory, Brain Plasticity Unit, CNRS, ESPCI Paris, PSL Research University, Paris, France

**Keywords:** Astrocyte, Alzheimer's disease, Metabolism, Drosophila, Long-term memory

## Abstract

Astrocytes help protect neurons from potential damage caused by reactive oxygen species (ROS). While ROS can also exert beneficial effects, it remains unknown how neuronal ROS signalling is activated during memory formation, and whether astrocytes play a role in this process. Here we discover an astrocyte-to-neuron H_2_O_2_ signalling cascade in *Drosophila* that is essential for long-term memory formation. Stimulation of astrocytes by acetylcholine induces an increase in intracellular calcium ions, which triggers the generation of extracellular superoxide (O_2_•^–^) by astrocytic NADPH oxidase. Astrocyte-secreted superoxide dismutase 3 (Sod3) converts O_2_•^–^ to hydrogen peroxide (H_2_O_2_), which is imported into neurons of the olfactory memory centre, the mushroom body, as revealed by in vivo H_2_O_2_ imaging. Notably, Sod3 activity requires copper ions, which are supplied by neuronal amyloid precursor protein. We also find that human amyloid-β peptide, implicated in Alzheimer’s disease, inhibits the nAChRα7 astrocytic cholinergic receptor and impairs memory formation by preventing H_2_O_2_ synthesis. These findings may have important implications for understanding the aetiology of Alzheimer’s disease.

## Abstract (web summary)

Rabah et al. discover an astrocyte-to-neuron hydrogen peroxide signalling cascade, which is crucial for long-term memory formation in *Drosophila*. This signalling is found to be inhibited by amyloid-β peptide, suggesting a link to Alzheimer’s disease.

## Main

Reactive oxygen species (ROS) such as superoxide (O_2_•^−^) and hydrogen peroxide (H_2_O_2_) are distinctive among metabolites because of their physiological ambivalence^1^. On the one hand, ROS are toxic byproducts that can irreversibly oxidize macromolecules, and powerful scavenging enzymes are active to protect cells from ROS damage. The production of ROS is closely linked to the activity of the mitochondria in the production of ATP, and neurons are particularly susceptible to ROS damage due to their high energy requirements and high content of unsaturated fatty acids that are prone to peroxidation^2^. On the other hand, ROS play a positive role by activating signalling pathways involved in physiological functions such as cell survival, growth or response to stress^1,2^. ROS signalling occurs mainly through reversible oxidation of redox-sensitive cysteines, which requires a sharply regulated spatiotemporal increase in ROS concentration^3^. In vitro studies of long-term potentiation in mice have shown that neuronal ROS signalling plays a positive role in synaptic plasticity^4,5^. Recent in vivo studies in *Drosophila* have shown that ROS signalling is required during development for activity-dependent plasticity in motoneurons^6,7^. While pioneering studies have reported that mutant mice with altered ROS metabolism display learning or memory defects^2,8^, it remains unknown how ROS signalling is recruited in vivo for memory formation. Astrocytes are known to regulate neuronal redox homeostasis and cognition in mice^9,10^. In particular, it was shown that under physiological conditions mitochondrial ROS in astrocytes stimulate nuclear factor erythroid-2-related factor 2 (NRF2) transcription factor expression, which promotes glutathione biosynthesis by astrocytes and antioxidant protection of neighbouring neurons^10^; however, it is not known whether neurons can trigger beneficial ROS production by astrocytes, and thereby if astrocytes are involved in the initiation of neuronal ROS signalling for synaptic plasticity.

Building a mechanistic view of ROS signalling during memory formation is essential not only from a brain physiology perspective, but it could also improve our understanding of the onset of Alzheimer’s disease (AD), a neurodegenerative disease characterized in particular by memory loss and defects in redox homeostasis^11,12^. At the neuropathological level, AD is characterized by the progressive formation in the brain of amyloid plaques that correspond to the extracellular accumulation of amyloid β (Aβ) peptide, generated by cleavage of the transmembrane amyloid precursor protein (APP), and by the formation of intracellular neurofibrillary tangles consisting of hyperphosphorylated tau protein^13^. Early synaptic dysfunction has been associated with AD^12,14^, which correlates with cognitive decline^15^; however, the cascade of events leading to this fatal condition remains unclear.

Here, we have addressed these key brain physiology and pathology issues in *Drosophila* by combining brain H_2_O_2_ imaging using recently developed roGFP2-Tsa2ΔC_R_ ultrasensitive sensors^16^ with functional characterization of ROS-related pathways during memory formation. We report that long-term memory (LTM) formation requires the establishment of a local H_2_O_2_ gradient in neurons of the olfactory memory centre, which follows the activation of ROS-producing enzymes in astrocytes. This signalling cascade is impaired by AD-related Aβ42. Our results provide a new framework to understand neuroglia interactions during memory formation in normal conditions and in AD.

## Results

### Astrocytes generate extracellular ROS for long-term memory

AD has been extensively linked to defects in redox homeostasis^11,12^. The objective of this study was to exploit the strengths of *Drosophila* genetics and in vivo imaging capabilities to further elucidate the relationships between Aβ, ROS and cognition; however, rather than artificially increasing ROS production and assessing its impact on memory formation, our approach was to investigate the potential beneficial effects of ROS on memory formation, with the understanding that this physiological pathway might be affected by Aβ in an AD-related model. Given the important role of neuron–glia metabolic coupling in *Drosophila* memory formation^17–19^, we sought to address these questions by investigating the potential role of astrocytes in neuronal ROS signalling.

**Fig. 1:**
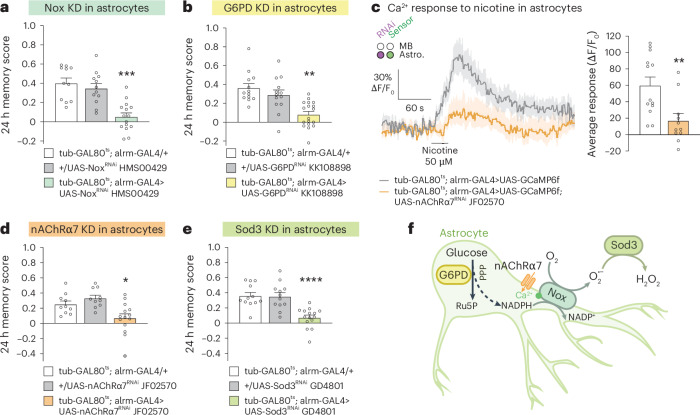
Astrocyte-derived H_2_O_2_ is required for LTM formation. **a**, *Nox* knockdown (KD) in adult astrocytes impaired memory after spaced conditioning (*n* = 11 (first group), *n* = 12 (second group), *n* = 15 (third group); F_2,35_ = 17.43, *P* = 0.000006). **b**, *G6PD* KD in adult astrocytes impaired memory after spaced conditioning (*n* = 13 (first and second groups), *n* = 18 (third group); F_2,41_ = 12.98, *P* = 0.00004). **c**, The fluorescent calcium sensor GCaMP6f was expressed in adult astrocytes. Nicotine stimulation (50 µM) elicited calcium increase in astrocytes, which was significantly decreased in *nAChRα7* KD (*n* = 13 (first group), *n* = 11 (second group), *t*_22_ = 3.15, *P* = 0.005). **d**, *nAChRα7* KD in adult astrocytes impaired memory after spaced conditioning (*n* = 10 (first and second groups), *n* = 14 (third group); F_2,31_ = 7.81, *P* = 0.002). **e**, *Sod3* KD in adult astrocytes impaired memory after spaced conditioning (*n* = 12 (first and second groups), *n* = 15 (third group); F_2,36_ = 16.02, *P* = 0.00001). **f**, Scheme of the molecular actors involved in astrocytes for LTM. Nox, activated by calcium entry through nAChRa7, produces superoxide O_2_•^−^ from O_2_ using the NADPH co-factor derived from the PPP. Extracellular O_2_•^−^ is converted to H_2_O_2_ by extracellular Sod3 secreted from the astrocytes. Ru5P, ribulose-5-phosphate. Data are presented as mean ± s.e.m. Genotype sample sizes are listed in the legend in order of bar appearance. Significance level of a two-sided *t*-test or the least significant Tukey pairwise comparisons following one-way analysis of variance (ANOVA). *P < 0.05; **P < 0.01; ***P < 0.001; ****P < 0.0001. Source data

We observed that inhibiting NADPH oxidase (Nox) expression in adult astrocytes impairs olfactory LTM in *Drosophila* (Fig. 1a). The only function of the transmembrane enzyme Nox is to produce extracellular O_2_•^−^ from O_2_ and intracellular NADPH^3^, and therefore our observation was potentially important from a ROS signalling perspective. For this experiment, we used a well-established paradigm of associative aversive olfactory conditioning^20,21^ named spaced conditioning (Methods), along with inducible and spatially controlled expression of a *Nox*-targeting RNAi. A single Nox enzyme exists in *Drosophila*, which shows strong sequence similarity with the human Nox5 (ref. ^22^). Similarly to Nox5 (ref. ^3^), *Drosophila* Nox is activable by Ca^2+^ thanks to intracellular EF-hands domains that bind Ca^2+^(ref. ^23^).

**Extended Data Fig. 1:**
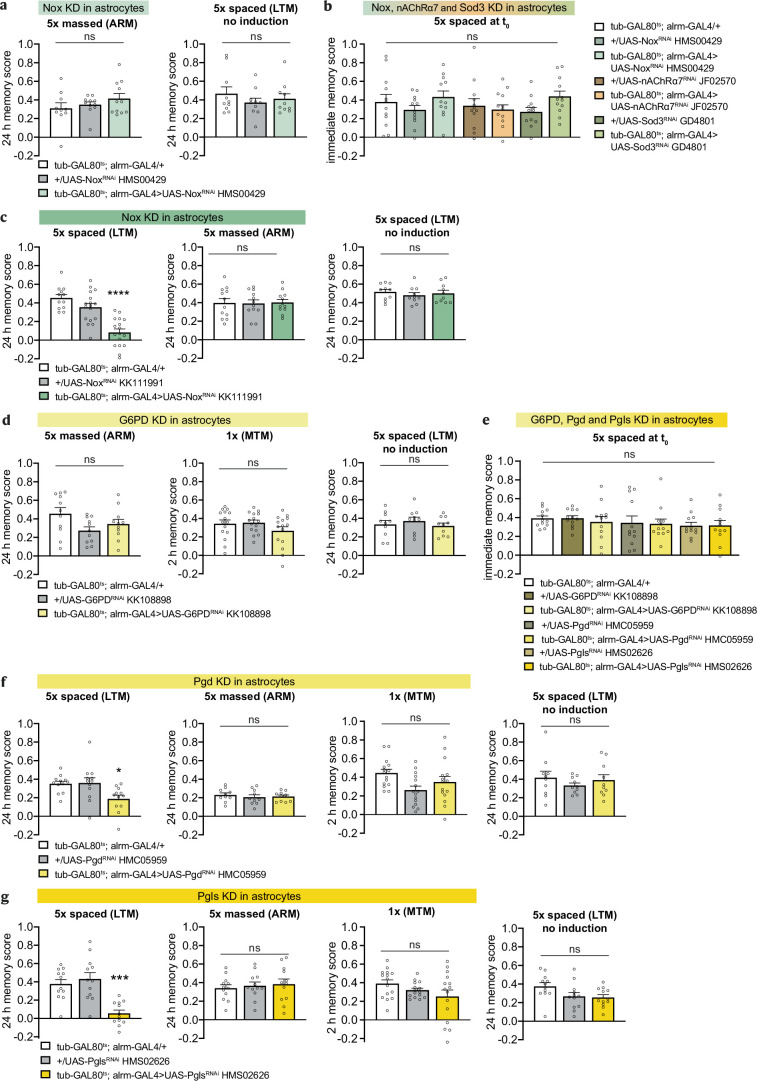
Controls for behavioural experiments, related to Fig. 1. (**a**) Memory after massed conditioning was not affected by *Nox* knockdown (KD) in adult astrocytes (n = 10 (first group), n = 11 (second and third groups), F_2,29_ = 1.17, P = 0.32). Without *Nox* RNAi induction, memory after spaced training was normal (n = 10, F_2,27_ = 0.67, P = 0.52). (**b**) Immediate memory after spaced conditioning was not affected by *Nox*, *nAChRα7*, or *Sod3* KD (n = 12, F_6,77_ = 1.233, P = 0.2989). (**c**) Memories at 24 h were assessed with a second RNAi against *Nox*. *Nox* KD impaired memory after spaced training (n = 12 (first group), n = 17 (second and third groups), F_2,43_ = 24.03, P = 0.00000009), but did not affect memory after massed training (n = 12, F_2,33_ = 0.018, P = 0.98). Without *Nox* RNAi induction, memory after spaced training was normal (n = 10, F_2,27_ = 0.38, P = 0.68). (**d**) Memory after massed conditioning was not affected by *G6PD* KD (n = 11, F_2,30_ = 2.93, P = 0.07). Memory at 2 h after 1x conditioning was not affected by *G6PD* KD (n = 15, F_2,42_ = 1.528, P = 0.2287). Without *G6PD* RNAi induction, memory after spaced training was normal (n = 10, F_2,27_ = 0.49, P = 0.62). (**e**) Immediate memory after spaced conditioning was not affected by *G6PD*, *Pgd* or *Pgls* KD (n = 12, F_6,77_ = 0.4174, P = 0.8653). (**f**) *Pgd* KD impaired memory after spaced training (n = 12, F_2,33_ = 4.92, P = 0.01), but did not affect memory after massed training (n = 10, F_2,27_ = 0.31, P = 0.73). Memory at 2 h after 1x conditioning was not affected by *Pgd* KD (n = 15, F_2,42_ = 3.582, P = 0.0366). Without *Pgd* RNAi induction, memory after spaced training was normal (n = 10, F_2,27_ = 0.61, P = 0.55). (**g**) *Pgls* KD impaired memory after spaced training (n = 12, F_2,33_ = 14.75, P = 0.00003), but did not affect memory after massed training (n = 12, F_2,33_ = 0.24, P = 0.79). Memory at 2 h after 1x conditioning was not affected by *Pgls* KD (n = 15, F_2,42_ = 2.078, P = 0.1379). Without *Pgls* RNAi induction, memory after spaced training was normal (n = 12, F_2,33_ = 3.11, P = 0.06). Data are presented as mean ± SEM. Sample sizes for each genotype are listed in the legend in the order of bar appearance. Significance level of the least significant Tukey pairwise comparison following one-way analysis of variance (ANOVA): *P < 0.05; ****P < 0.0001; ns: not significant, P > 0.05. Source data

LTM formation requiring Nox activity in astrocytes depends on de novo protein synthesis^21^ in neurons of the olfactory memory centre, the mushroom body (MB)^24,25^. Conversely, anaesthesia-resistant memory (ARM), which forms after massed conditioning (Methods) and does not depend on de novo protein synthesis^21^, was not affected by *Nox* knockdown in adult astrocytes (Extended Data Fig. 1a). In addition, *Nox* knockdown did not affect perception of the stimuli used for conditioning (Supplementary Table 1), and LTM was normal when *Nox* RNAi expression was not induced (Extended Data Fig. 1a). To further ensure the specificity of Nox implication in LTM, we measured the immediate memory capacity after spaced conditioning of flies expressing *Nox* RNAi in adult astrocytes. Our results demonstrated that *Nox* knockdown did not affect immediate memory, confirming that superoxide production by astrocytes is required specifically for LTM, as measured at 24 h after spaced conditioning (Extended Data Fig. 1b). Last, we confirmed the involvement of Nox in adult astrocytes for LTM with a second nonoverlapping *Nox* RNAi (Extended Data Fig. 1c and Supplementary Table 1). These controls were performed in a systematic manner throughout the course of this study. With regard to text simplification, knockdown flies exhibiting the aforementioned phenotypes (impaired LTM as measured at 24 h after conditioning, assessed with two RNAis; normal ARM as measured at 24 h after massed conditioning; normal immediate memory after spaced conditioning; normal stimuli perception; normal LTM in the absence of RNAi induction) will be simply reported as displaying a specific LTM defect.

The Nox substrate NADPH is mainly produced by the pentose phosphate pathway (PPP)^26^. To study this pathway we inhibited in adult astrocytes the expression of G6PD, the enzyme catalysing the first step of the PPP^26^, and we observed a specific LTM defect (Fig. 1b, Extended Data Fig. 1d,e and Supplementary Table 1). Given that the PPP generates many intermediates involved in various biosynthetic processes, including fatty acids and nucleotides^26^, an additional experiment was conducted to ensure that inhibiting the PPP in astrocytes did not affect different forms of olfactory memory in an unspecific manner. Thus, in addition to the assessment of ARM following massed conditioning and immediate memory following spaced conditioning, the middle-term memory capacity following a single conditioning cycle was evaluated after *G6PD* knockdown in adult astrocytes. *G6PD* knockdown flies displayed normal middle-term memory (Extended Data Fig. 1d). The essential role of astrocytic PPP in LTM formation was confirmed by inhibiting the expression in astrocytes of Pgd and Pgls^26^, two other enzymes in this pathway (Extended Data Fig. 1e–g and Supplementary Table 1). Altogether, these results support the notion that astrocytic Nox generates O_2_•^−^ required for LTM formation.

**Extended Data Fig. 2:**
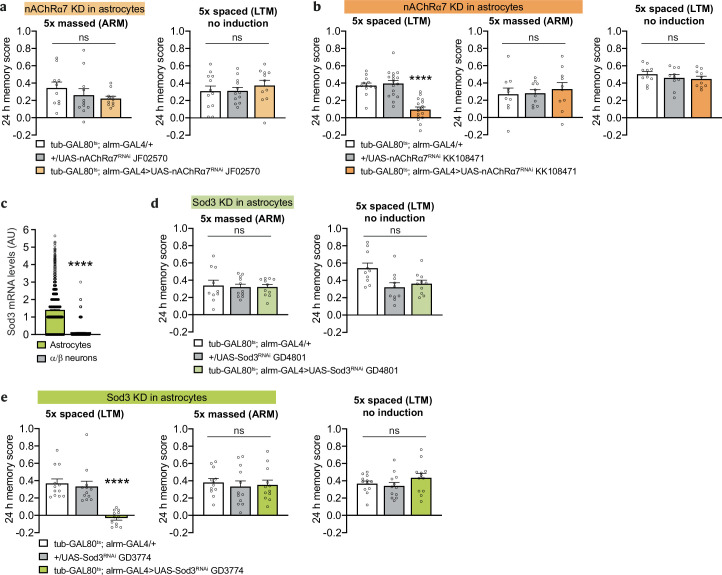
Controls for behavioural experiments, related to Fig. 1. (**a**) Memory after massed conditioning was not affected by *nAChRα7* KD in adult astrocytes (n = 10 (first group), n = 11 (second and third groups), F_2,29_ = 1.07, P = 0.36). Without *nAChRα7* RNAi induction, memory after spaced training was normal (n = 12 (first and second groups), n = 11 (third group), F_2,32_ = 0.47, P = 0.63). (**b**) Memories at 24 h were assessed with a second RNAi against *nAChRα7*. *nAChRα7* KD in adult astrocytes impaired memory after spaced training (n = 13 (first group), n = 17 (second and third groups), F_2,44_ = 26.95, P = 0.00000002), but did not affect memory after massed training (n = 10, F_2,27_ = 0.24, P = 0.79). Without *nAChRα7* RNAi induction, memory after spaced training was normal (n = 10, F_2,27_ = 0.71, P = 0.50). (**c**) Relative expression of *Sod3* in astrocytes and α/β MB neurons according to published single-cell transcriptomics^33^. Levels of *Sod3* mRNA are higher in astrocytes as compared to α/β MB neurons (n = 1407 (first group), n = 672 (second group), t_2077_ = 28.04, P < 0.000000000000001). (**d**) Memory after massed conditioning was not affected by *Sod3* KD in adult astrocytes (n = 10 (first group), n = 11 (second and third groups), F_2,29_ = 0.06, P = 0.94). Without *Sod3* RNAi induction, memory after spaced training was normal (n = 10, F_2,27_ = 5.15, P = 0.01). (**e**) Memories at 24 h were assessed with a second RNAi against *Sod3*. *Sod3* KD in adult astrocytes impaired memory after spaced training (n = 12, F_2,33_ = 20.88, P = 0.000001), but did not affect memory after massed training (n = 12, F_2,33_ = 0.19, P = 0.83). Without *Sod3* RNAi induction, memory after spaced training was normal (n = 12, F_2,33_ = 1.35, P = 0.27). Data are presented as mean ± SEM. Sample sizes for each genotype are listed in the legend in the order of bar appearance. Significance level of a two-sided t-test or the least significant Tukey pairwise comparison following one-way analysis of variance (ANOVA): ****P < 0.0001; ns: not significant, P > 0.05. Source data

As stated above, similar to the human Nox5, *Drosophila* Nox is activated by Ca^2+^. Acetylcholine (ACh) is the major excitatory neurotransmitter of *Drosophila* brain neurons, and in particular MB neurons are cholinergic^27^. Among the ionotropic cholinergic receptors, the channel formed of nAChRα7 subunits is the most permeable to Ca^2+^(ref. ^28^) and is expressed in human astrocytes^29^. To examine whether nAChRα7 cholinergic stimulation induces a Ca^2+^ flux in *Drosophila* astrocytes, we expressed the GCaMP6f Ca^2+^ fluorescent reporter in astrocytes and stimulated the brains of live flies with nicotine, an nAChRα7 agonist. Nicotine stimulation induced an increased astrocytic Ca^2+^ concentration in control flies (Fig. 1c). This effect was dampened when *nAChRα7* expression was inhibited in astrocytes (Fig. 1c). We next addressed the role of astrocytic nAChRα7 in memory and showed that *nAchRα7* knockdown in adult astrocytes specifically impaired LTM (Fig. 1d, Extended Data Figs. 1b and 2a,b and Supplementary Table 1). These results suggest that during spaced conditioning ACh release can activate Ca^2+^ signalling in astrocytes through nAChRα7 and is required for LTM. Consistent with our findings, astrocytic nAChRα7 was implicated in the persistence of fear memory in mice^30^.

While O_2_•^−^ is a highly reactive and therefore toxic ROS with a short half-life, H_2_O_2_, which is less reactive and has a longer half-life, is more suitable for ROS signalling^1,31^. Astrocytic Nox activity at the plasma membrane produces extracellular O_2_•^−^, whose conversion into H_2_O_2_ is catalysed by the only secreted superoxide dismutase (Sod), Sod3 (ref. ^32^). In the *Drosophila* brain, *Sod3* is more strongly expressed in astrocytes than in neurons, as shown by single-cell transcriptomics data^33^ (Extended Data Fig. 2c). *Sod3* knockdown in adult astrocytes specifically impaired LTM (Fig. 1e and Extended Data Figs. 1b and 2d,e and Supplementary Table 1). Altogether, these results suggest that a nAChRα7–Nox–Sod3 signalling cascade takes place upon LTM formation, which is triggered by ACh stimulation of astrocytes, resulting in extracellular H_2_O_2_ formation (Fig. 1f).

Like the tripartite synapse in the mammalian brain^34^, astrocytic processes overlap with synapses in the *Drosophila* brain^35^. We therefore wondered whether astrocytes might be a source of beneficial ROS imported by neurons during LTM formation. In particular, is H_2_O_2_ signalling involved during LTM formation in the MB?

### An H_2_O_2_ gradient in MB α lobe supports LTM formation

**Fig. 2:**
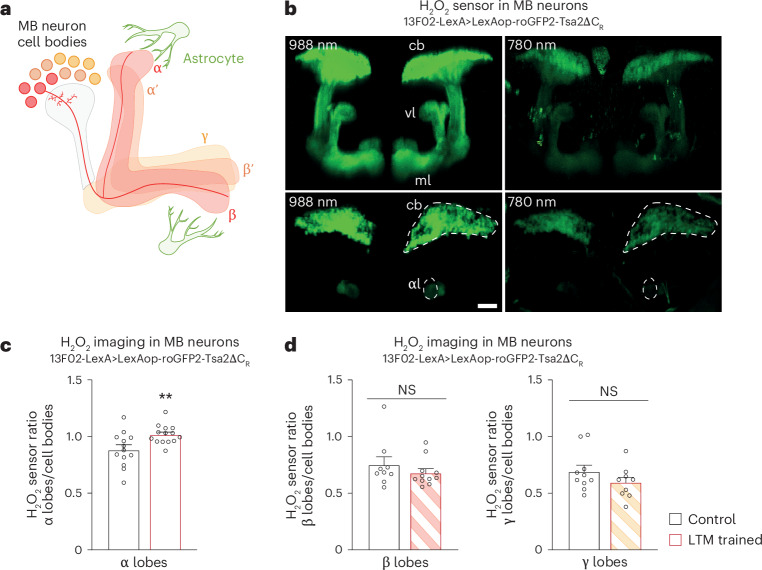
An H_2_O_2_ gradient forms in MB α lobes upon LTM formation. **a**, Illustration of MB structure. Axons of MB neurons follow a branching pattern that forms vertical lobes (α and α′) and medial lobes (β, β′ and γ). **b**, Images of the roGFP2-Tsa2ΔC_R_ H_2_O_2_ sensor expressed in MB neurons. 3D reconstructions of image stacks (128-µm-thick stack, 1 µm Z-step) of the H_2_O_2_ sensor in MB neurons at the two excitation wavelengths (988 and 780 nm) (top). Cb, cell bodies; vl, vertical lobes (α and α′); ml, medial lobes (β, β′ and γ). Horizontal plane with regions of interest, cell bodies and α vertical lobes delimited with dashed lines (bottom). Scale bar, 30 µm. **c**, The H_2_O_2_ level in α lobes normalized to the cell bodies value is increased upon spaced training, in comparison to the non-associative unpaired control (*n* = 13 (first group), *n* = 14 (second group); *t*_25_ = 2.82, *P* = 0.009). **d**, The H_2_O_2_ level in β or γ lobes normalized to the H_2_O_2_ level in cell bodies after spaced training was similar to the unpaired control (β, *n* = 9 (first group), *n* = 11 (second group), *t*_18_ = 0.94, *P* = 0.36; γ, *n* = 10 (first group), *n* = 9 (second group), *t*_17_ = 1.30, *P* = 0.21). Data are presented as mean ± s.e.m. Genotype sample sizes are listed in the legend in order of bar appearance. Significance level of a two-sided *t*-test. **P < 0.01; NS, not significant, *P* > 0.05. Source data

**Extended Data Fig. 3:**
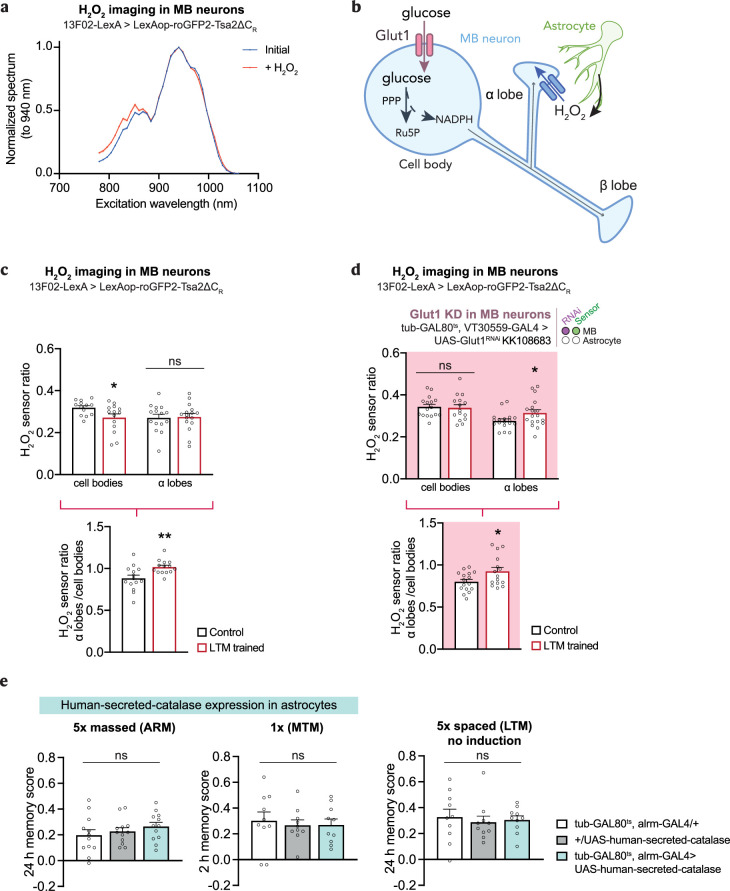
roGFP2-Tsa2ΔC_R_ imaging in MB neurons, related to Figs. 2 and 3. (**a**) Fluorescence excitation spectra of roGFP2-Tsa2ΔC_R_ upon 2-photon excitation in basal and oxidized states. H_2_O_2_-induced oxidation of the probe led to an increase in the excitation spectrum at 780 nm and a slight decrease at 988 nm, allowing ratiometric measurement of the oxidation level of the probe (ratio 780 nm/988 nm). (**b**) Intracellular pathways recruited during LTM formation in MB neurons. Upon spaced training, glucose intake in MB neuron cell bodies via the Glut1 transporter is increased to fuel the PPP, which produces reductive power in the form of NADPH^26^. (**c**) Measurements of H_2_O_2_ levels in cell bodies and α lobes using the roGFP2-Tsa2ΔC_R_ H_2_O_2_ sensor expressed in MB neurons. H_2_O_2_ level (measured as 780/988 nm ratio) decreased in cell bodies upon spaced training (n = 13 (first group), n = 14 (second group), t_25_ = 2.27, P = 0.03), while it did not vary in α lobes (n = 15 (first group), n = 16 (second group), t_29_ = 0.18, P = 0.86). The H_2_O_2_ level in α lobes normalized to the cell bodies value is increased upon spaced training in adult MB neurons (values, bottom panel, same value as in Fig. 2c). (**d**) *Glut1* KD in MB neurons abolished the decrease in H_2_O_2_ level in cell bodies (n = 16 (first group), n = 15 (second group), t_29_ = 0.25, P = 0.81, top panel), revealing an increase in H_2_O_2_ level in α lobes after spaced training (n = 18, t_34_ = 2.06, P = 0.047, top panel). The H_2_O_2_ level in α lobes normalized to the cell bodies value is increased upon spaced training when *Glut1* was knocked down in adult MB neurons (n = 16 (first group), n = 15 (second group), t_29_ = 2.66, P = 0.013, bottom panel). (**e**) Memory after massed conditioning was not affected by human-secreted-catalase expression in astrocytes (n = 12, F_2,33_ = 1.026, P = 0.9116). Memory at 2 h after 1x conditioning was not affected by human-secreted-catalase expression in adult astrocytes (n = 10, F_2,27_ = 1.171, P = 0.1304). Without human-secreted-catalase induction, memory after spaced training was normal (n = 10, F_2,27_ = 1.539, P = 0.8436). Data are presented as mean ± SEM. Sample sizes for each genotype are listed in the legend in the order of bar appearance. Significance level of a two-sided t-test or the least significant Tukey pairwise comparison following one-way analysis of variance (ANOVA): *P < 0.05; **P < 0.01; ns: not significant, P > 0.05. Source data

The MB is a bilateral structure consisting of about 2,000 cholinergic neurons^27^ in each brain hemisphere. Axons of the MB neurons form bundles and branch out, giving shape to anatomical structures called lobes that synapse with the MB output neurons. MB neurons are classified into three different subtypes: the α/β neurons, whose axons branch to form an α vertical projection and a medial β projection (Fig. 2a); the α′/β′ neurons; and the γ neurons, which form a single medial γ lobe. The axonal projection of the α lobe is specifically involved in LTM formation^20,36^. We aimed to image H_2_O_2_ in MB neurons upon LTM formation. Recently, the ability to observe physiological H_2_O_2_ levels has become possible with the development of an ultrasensitive H_2_O_2_ fluorescent sensor, roGFP2-Tsa2ΔC_R_^16^, which we imported and characterized in *Drosophila* (Fig. 2b and Extended Data Fig. 3a).

To observe change in H_2_O_2_ dynamics, flies expressing the H_2_O_2_ sensor in the MB were imaged between 0.5 h and 2 h after spaced conditioning, a time window when early LTM-encoding events occur^17,37^. Control flies were submitted to non-associative unpaired protocol that does not allow memory formation. Notably, in flies trained for LTM, we observed an increased H_2_O_2_ level in the MB α lobe (Fig. 2c), and no increase in the MB β and γ medial lobes (Fig. 2d). An additional point must be considered to interpret the H_2_O_2_ imaging data. As we previously demonstrated, *Drosophila* LTM formation involves activation of the PPP in MB neurons, fuelled by Glut1-mediated glucose import^17^. As PPP activity generates the reducing agent NADPH, its activation in the MB is expected to result in a global decrease in ROS concentration (compare with scheme in Extended Data Fig. 3b). Indeed, detailed examination of imaging data revealed a decreased H_2_O_2_ level in MB cell bodies in flies trained for LTM (Extended Data Fig. 3c). Notably, when glucose import was impaired in adult MB, the decreased H_2_O_2_ response in MB neuron cell bodies after spaced conditioning was abolished, but the H_2_O_2_ increase was observed in the α lobe (Extended Data Fig. 3d). Altogether, these data reveal the existence in LTM-forming flies of an H_2_O_2_ gradient within the MB neurons, generated by H_2_O_2_ influx within the α vertical lobe. These results are in agreement with the fact that the α lobe is specifically involved in LTM formation^20,36^.

**Fig. 3:**
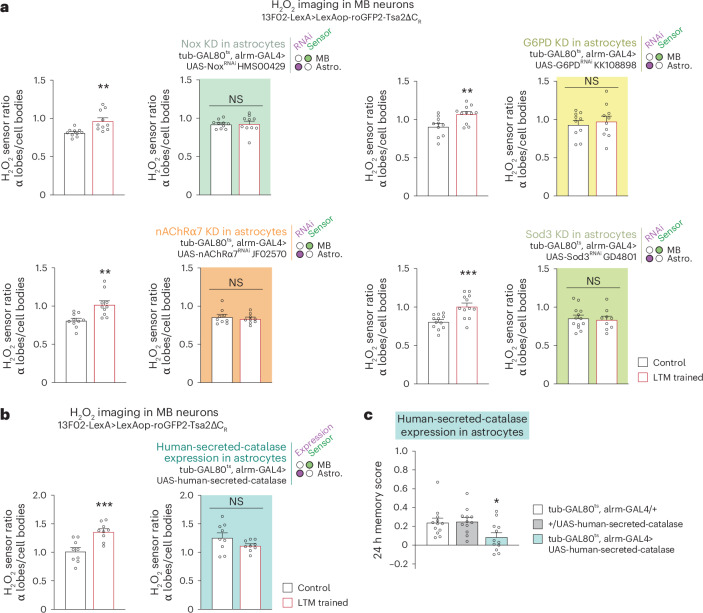
Astrocytes are a source of H_2_O_2_ imported into MB α lobes. **a**, Measurements of H_2_O_2_ levels in MB neurons after spaced training in the context of astrocytic KDs. The increase in the α lobes/cell bodies H_2_O_2_ level elicited by spaced training was impaired in KD of *Nox* (*n* = 10, *t*_18_ = 0.11, *P* = 0.91; wild-type control: *n* = 10, *t*_18_ = 3.47, *P* = 0.003), *G6PD* (*n* = 10 (first and second groups), *t*_18_ = 0.49, *P* = 0.63; wild-type control: *n* = 10 (first group), *n* = 11 (second group), *t*_19_ = 3.01, *P* = 0.007), *nAChRα7* (*n* = 9, *t*_16_ = 0.63, *P* = 0.54; wild-type control: *n* = 10, t_18_ = 3.57, *P* = 0.002) and *Sod3* (*n* = 13 (first group), *n* = 9 (second group), *t*_20_ = 0.33, *P* = 0.75; wild-type control: *n* = 12, *t*_22_ = 3.93, *P* = 0.0007). Data are presented as mean ± s.e.m. **b**, Expression of human-secreted-catalase in astrocytes abolished the increase in the α lobes/cell bodies H_2_O_2_ level elicited by spaced training (*n* = 9, *t*_16_ = 1.596, *P* = 0.13; wild-type control: *n* = 9, *t*_16_ = 4.136, *P* = 0.0008). **c**, Expression of human-secreted-catalase in astrocytes impaired 24 h memory after spaced conditioning (*n* = 12, *F*_(2,33)_ = 4.603, *P* = 0.017). Data are presented as mean ± s.e.m. Genotype sample sizes are listed in the legend in order of bar appearance. Significance level of two-sided *t*-test or the least significant Tukey pairwise comparison following one-way ANOVA. *P < 0.05; **P < 0.01; ***P < 0.001; NS, not significant, *P* > 0.05. Source data

To test whether astrocyte activity modulates H_2_O_2_ signalling in the MB LTM centre, we imaged H_2_O_2_ in MB neurons while interfering with the H_2_O_2_-producing cascade in astrocytes. The increased level of H_2_O_2_ in the MB α lobe was lost by inhibiting the expression of either *Nox*, *nAChRα7*, *G6PD* or *Sod3* in adult astrocytes (Fig. 3a). These results indicate that the H_2_O_2_ gradient formed in MB neurons after spaced conditioning originates from astrocytic Nox and Sod3 activity in response to ACh stimulation. To substantiate this hypothesis, we intended to deplete the extracellular H_2_O_2_ generated by astrocytic Sod3 during LTM formation by expressing a secreted form of the human catalase in astrocytes^7^. Catalase is responsible for catalysing the breakdown of H_2_O_2_ into H_2_O and O_2_. Notably, the typical increase in H_2_O_2_ observed in the α lobe following spaced conditioning was absent in the presence of the secreted catalase (Fig. 3b). In accordance with the imaging data, the LTM of flies that expressed the secreted catalase was found to be specifically impaired (Fig. 3c, Extended Data Fig. 3e and Supplementary Data Table 2). These findings substantiate the hypothesis that the H_2_O_2_ gradient observed in the MB during LTM formation is due to extracellular SOD3 activity.

### A redox relay cascade is required in MB for LTM formation

**Fig. 4:**
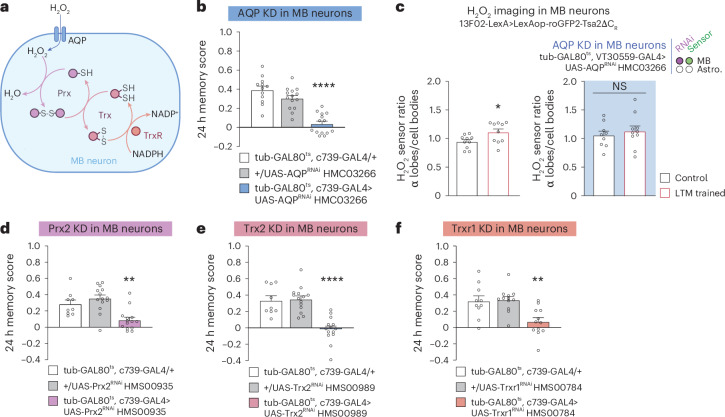
A redox-sensitive cascade is recruited upon H_2_O_2_ entry into MB neurons for LTM formation. **a**, Putative redox relay cascade downstream of H_2_O_2_ import in MB neurons. **b**, *AQP* KD in adult α/β MB neurons impaired memory after spaced conditioning (*n* = 12 (first group), *n* = 14 (second and third groups), *F*_2,37_ = 31.24, *P* = 0.00000001). **c**, The increase in H_2_O_2_ level in α lobes/cell bodies elicited by spaced training (*n* = 10, *t*_18_ = 2.42, *P* = 0.0261) was impaired in *AQP* KD in adult MB neurons (*n* = 9 (first group), *n* = 10 (second group), *t*_17_ = 0.59, *P* = 0.56). **d**, *Prx2* KD in adult α/β MB neurons impaired memory after spaced conditioning (*n* = 9 (first group), *n* = 14 (second and third groups), *F*_2,34_ = 12.08, *P* = 0.0001). **e**, *Trx2* KD in adult α/β MB neurons impaired memory after spaced conditioning (*n* = 9 (first group), *n* = 14 (second and third groups), *F*_2,37_ = 14.43, *P* = 0.0000007). **f**, *Trxr1* KD in adult α/β MB neurons impaired memory after spaced conditioning (*n* = 10 (first group), *n* = 12 (second and third groups), *F*_2,27_ = 20.30, *P* = 0.0012). Data are presented as mean ± s.e.m. Genotype sample sizes are listed in the legend in order of bar appearance. Significance level of a two-sided *t*-test or the least significant Tukey pairwise comparison following one-way ANOVA. *P < 0.05; **P < 0.01; ****P < 0.0001; NS, not significant, *P* > 0.05. Source data

**Extended Data Fig. 4:**
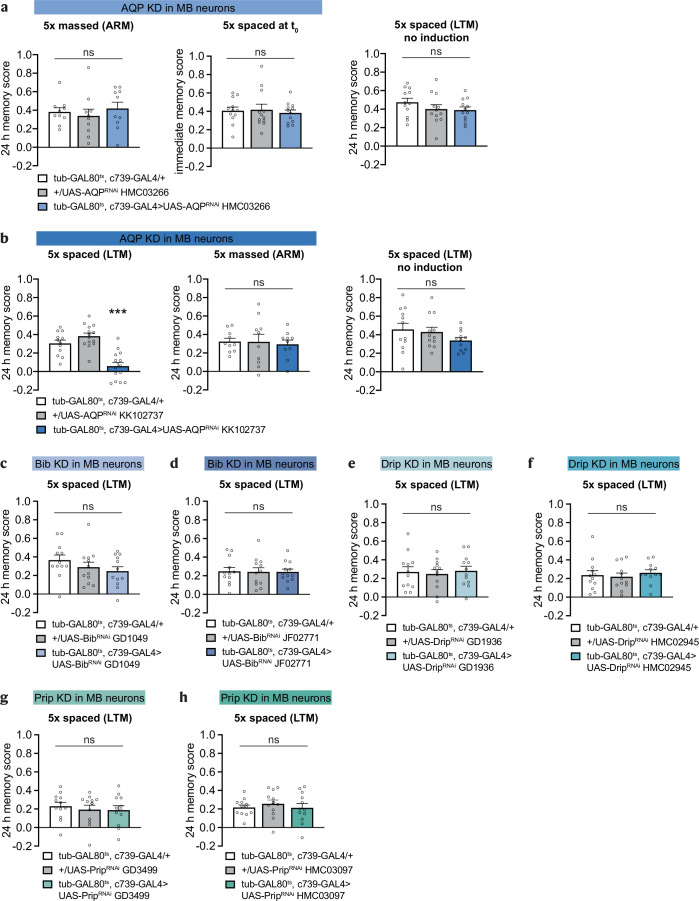
Controls for behavioural experiments, and analysis of alternative Drosophila aquaporins, related to Fig. 4. (**a**) Memory after massed conditioning was not affected by *AQP* KD in adult α/β MB neurons (n = 10, F_2,27_ = 0.40, P = 0.68). Immediate memory after spaced conditioning was not affected by *AQP* KD in adult MB neurons (n = 12, F_2,33_ = 0.1461, P = 0.8647). Without *AQP* RNAi induction, memory after spaced training was normal (n = 12, F_2,33_ = 1.20, P = 0.32). (**b**) Memories at 24 h were assessed with a second RNAi against *AQP*. *AQP* KD in adult α/β MB neurons impaired memory after spaced training (n = 12 (first group), n = 14 (second and third groups), F_2,37_ = 21.55, P = 0.0000006), but did not affect memory after massed training (n = 10, F_2,27_ = 0.08, P = 0.92). Without *AQP* RNAi induction, memory after spaced training was normal (n = 12, F_2,33_ = 1.40, P = 0.26). (**c**) and (**d**) *Bib* KD in adult α/β MB neurons did not impair memory after spaced training (c: n = 12, F_2,33_ = 1.327, P = 0.2791; d: n = 12, F_2,33_ = 0.0081, P = 0.99). (**e**) and (**f**) *Drip* KD in adult α/β MB neurons did not impair memory after spaced training (e: n = 12, F_2,33_ = 0.1146, P = 0.8921; f: n = 12, F_2,33_ = 0.2708, P = 0.7644). (**g**) and (**h**) *Prip* KD in adult α/β MB neurons did not impair memory after spaced training (g: n = 12, F_2,33_ = 0.2466, P = 0.7829; h: n = 12, F_2,33_ = 0.3601, P = 0.7003). Data are presented as mean ± SEM. Sample sizes for each genotype are listed in the legend in the order of bar appearance. Significance level of the least significant Tukey pairwise comparison following one-way analysis of variance (ANOVA): ****P < 0.0001; ns: not significant, P > 0.05. Source data

How does H_2_O_2_ produced extracellularly by astrocytic enzymes enter MB neurons upon LTM formation? Although H_2_O_2_ can diffuse across the plasma membrane, H_2_O_2_ import is strongly facilitated by channels of the aquaporin protein family^38^. We tested the potential involvement of the main *Drosophila* aquaporin, AQP, in LTM (Fig. 4a). *AQP* knockdown in adult MB α/β neurons specifically impaired LTM (Fig. 4b, Extended Data Fig. 4a,b and Supplementary Table 3). The LTM defect following *AQP* knockdown was linked to a loss of the H_2_O_2_ increase in the α lobe after spaced conditioning (Fig. 4c). Three additional aquaporin orthologues have been identified in *Drosophila*: drip, bib and prip^7^. The inhibition of their expression in adult α/β MB neurons did not affect LTM (Extended Data Fig. 4c–h), indicating that AQP plays a specific role in H_2_O_2_ import following SOD3 activity.

**Extended Data Fig. 5:**
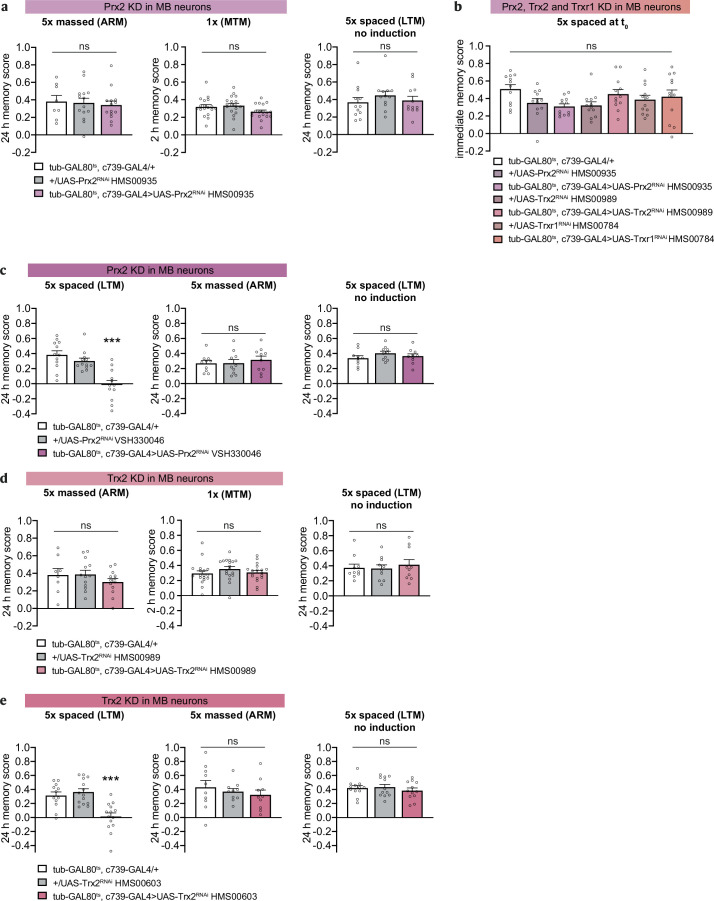
Controls for behavioural experiments, related to Fig. 4. (**a**) Memory after massed conditioning was not affected by *Prx2* KD in adult α/β MB neurons (n = 8 (first group), n = 14 (second and third groups), F_2,33_ = 0.13, P = 0.88). Memory at 2 h after 1x conditioning was not affected by *Prx2* KD in adult α/β MB (n = 12, F_2,51_ = 2.288, P = 0.1118). Without *Prx2* RNAi induction, memory after spaced training was normal (n = 12, F_2,33_ = 0.64, P = 0.53). (**b**) Immediate memory after spaced conditioning was not affected by *Prx2*, *Trx2* or *Trxr1* KD in adult α/β MB (n = 12, F_6,77_ = 1.958, P = 0.0821). (**c**) Memories at 24 h were assessed with a second RNAi against *Prx2*. *Prx2* KD in adult α/β MB neurons impaired memory after spaced training (n = 12, F_2,33_ = 16.48, P = 0.00001), but did not affect memory after massed training (n = 10, F_2,27_ = 0.33, P = 0.73). Without *Prx2* RNAi induction, memory after spaced training was normal (n = 10, F_2,27_ = 1.09, P = 0.35). (**d**) Memory after massed conditioning was not affected by *Trx2* KD in adult α/β MB neurons (n = 8 (first group), n = 13 (second and third groups), F_2,31_ = 0.99, P = 0.38). Memory at 2 h after 1x conditioning was not affected by *Trx2* KD in adult α/β MB (n = 18, F_2,51_ = 0.9106, P = 0.4087). Without *Trx2* RNAi induction, memory after spaced training was normal (n = 10, F_2,27_ = 0.23, P = 0.80). (**e**) Memories at 24 h were assessed with a second RNAi against *Trx2*. *Trx2* KD in adult α/β MB neurons impaired memory after spaced training (n = 12 (first group), n = 14 (second and third groups), F_2,37_ = 14.43, P = 0.00002), but did not affect memory after massed training (n = 10, F_2,27_ = 0.54, P = 0.59). Without *Trx2* RNAi induction, memory after spaced training was normal (n = 12, F_2,33_ = 0.47, P = 0.63). Data are presented as mean ± SEM. Sample sizes for each genotype are listed in the legend in the order of bar appearance. Significance level of the least significant Tukey pairwise comparison following one-way analysis of variance (ANOVA): ***P < 0.001; ns: not significant, P > 0.05. Source data

**Extended Data Fig. 6:**
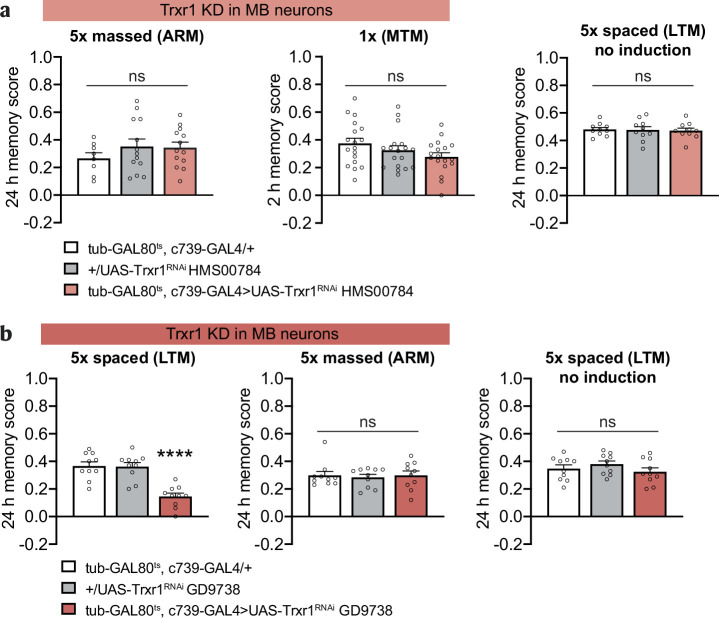
Complementary controls for behavioural experiments, related to Fig. 4. (**a**) Memory after massed conditioning was not affected by *Trxr1* KD in adult α/β MB neurons (n = 8 (first group), n = 13 (second and third groups), F_2,31_ = 0.81, P = 0.45). Memory at 2 h after 1x conditioning was not affected by *Trxr1* KD in adult α/β MB (n = 18, F_2,51_ = 2.075, P = 0.1361). Without *Trxr1* RNAi induction, memory after spaced training was normal (n = 10, F_2,27_ = 0.05, P = 0.95). (**b**) Memories at 24 h were assessed with a second RNAi against *Trxr1*. *Trxr1* KD in adult α/β MB neurons impaired memory after spaced training (n = 10, F_2,27_ = 20.30, P = 0.000004), but did not affect memory after massed training (n = 10, F_2,27_ = 0.09, P = 0.92). Without *Trxr1* RNAi induction, memory after spaced training was normal (n = 10, F_2,27_ = 1.07, P = 0.36). Data are presented as mean ± SEM. Sample sizes for each genotype are listed in the legend in the order of bar appearance. Significance level of the least significant Tukey pairwise comparison following one-way analysis of variance (ANOVA): ****P < 0.0001; ns: not significant, P > 0.05. Source data

Our results support the idea that H_2_O_2_ signalling in α/β neurons occurs for LTM formation. H_2_O_2_ signalling is primarily characterized by the reversible oxidation of target cysteines on signalling proteins, which subsequently modifies their activity. This is achieved either by direct action of H_2_O_2_ following an increase in local concentration, or through a cascade of redox-sensitive relay enzymes, starting with the Cys-based peroxidase peroxiredoxin (Prx), an H_2_O_2_ scavenger (Fig. 4a)^39,40^. We identified Prx2 as the major Prx involved in LTM formation (Fig. 4d, Extended Data Fig. 5a–c and Supplementary Table 4). Oxidized Prx are reduced by thioredoxin (Trx)^40^, and we showed that *Drosophila* Trx2 is specifically involved in LTM (Fig. 4e, Extended Data Fig. 5b,d,e and Supplementary Table 4). Last, Trx is reduced by a Trx reductase (Trxr) that oxidizes NADPH into NADP^+^ in the process. We identified Trxr1 as a major actor of LTM formation in α/β neurons (Fig. 4f, Extended Data Figs. 5b and 6a,b and Supplementary Table 4). Prx H_2_O_2_ scavenging enzymes are involved in the general maintenance of redox homeostasis^39,40^. Consequently, the inhibition of Prx2 expression may potentially impact α/β MB neurons in an unspecific manner. To eliminate this possibility, we conducted an additional experiment, measuring middle-term memory after a single conditioning cycle of *Prx2*, *Trx2* or *Trxr1* knockdowns. This control is of particular significance given that middle-term memory is encoded by α/β neurons, as is LTM^41^. Knocking down *Prx2*, *Trx2* or *Trxr1* in α/β neurons did not affect middle-term memory, demonstrating the specificity of the effect on LTM (Extended Data Figs. 5a,d and 6a).

Altogether, these results reveal a cascade of redox-sensitive enzymes required for LTM formation in α/β MB neurons. This cascade could be involved in the regulation of gene expression required for LTM formation^1,21^.

### Appl delivers extracellular copper for SOD3 activity

**Fig. 5:**
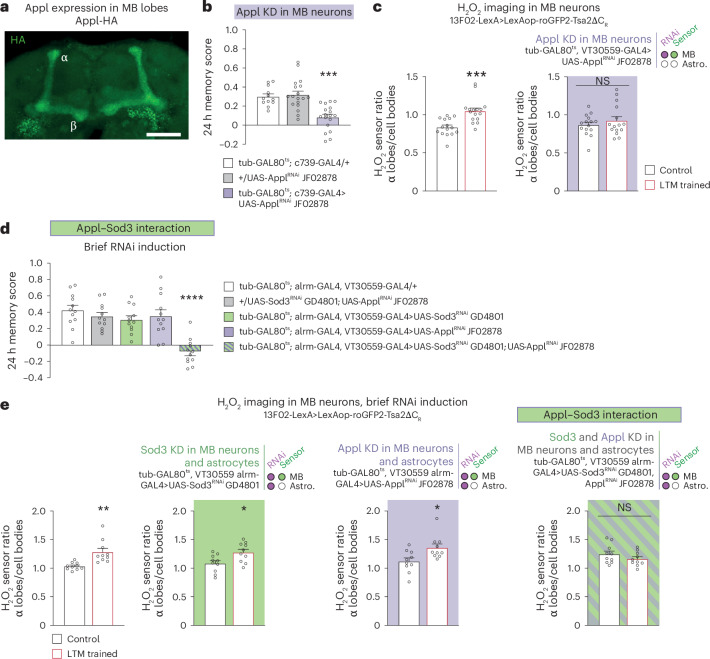
*Appl* interacts with *Sod3* during LTM formation. **a**, Image (maximum intensity projection of 22-µm-thick Z-stack acquisition, Z-step 1 µm) of C-terminal HA-tagged Appl immunostaining showing the high expression of HA-tagged Appl in α/β MB neurons (green). Scale bar, 40 µm. **b**, *Appl* KD in adult α/β neurons impaired memory after spaced conditioning (*n* = 12 (first group), 18 (second and third groups), *F*_2,45_ = 15.58, *P* = 0.000007). **c**, The increase in H_2_O_2_ level in α lobes/cell bodies elicited by spaced training (*n* = 15 (first group), *n* = 16 (second group), *t*_29_ = 4.30, *P* = 0.0002) was impaired in *Appl* KD in adult MB neurons (*n* = 15, *t*_28_ = 0.88, *P* = 0.39). **d**, Mild decreases in both *Appl* and *Sod3* expression in MB neurons and astrocytes impaired memory after spaced conditioning (*n* = 12, *F*_4,55_ = 13.22, *P* = 0.0000001). This mild decrease was obtained by activating RNAi expression for 1 day instead of 3 days. **e**, Mild decrease in both *Appl* and *Sod3* expression in MB neurons and astrocytes prevents increase in H_2_O_2_ level elicited by spaced training (wild-type control: *n* = 10, *t*_18_ = 3.741, *P* = 0.0015; *Sod3* KD: *n* = 10, *t*_18_ = 2.651, *P* = 0.0163; *Appl* KD: *n* = 10, *t*_18_ = 2.655, *P* = 0.0161; *Sod3* and *Appl* KD: *n* = 10, *t*_18_ = 1.262, *P* = 0.2229). Data are presented as mean ± s.e.m. Genotype sample sizes are listed in the legend in order of bar appearance. Significance level of a two-sided *t*-test or the least significant pairwise Tukey comparison following one-way ANOVA. *P < 0.05; **P < 0.01; ***P < 0.001; ****P < 0.0001; NS, not significant, *P* > 0.05. Source data

**Extended Data Fig. 7:**
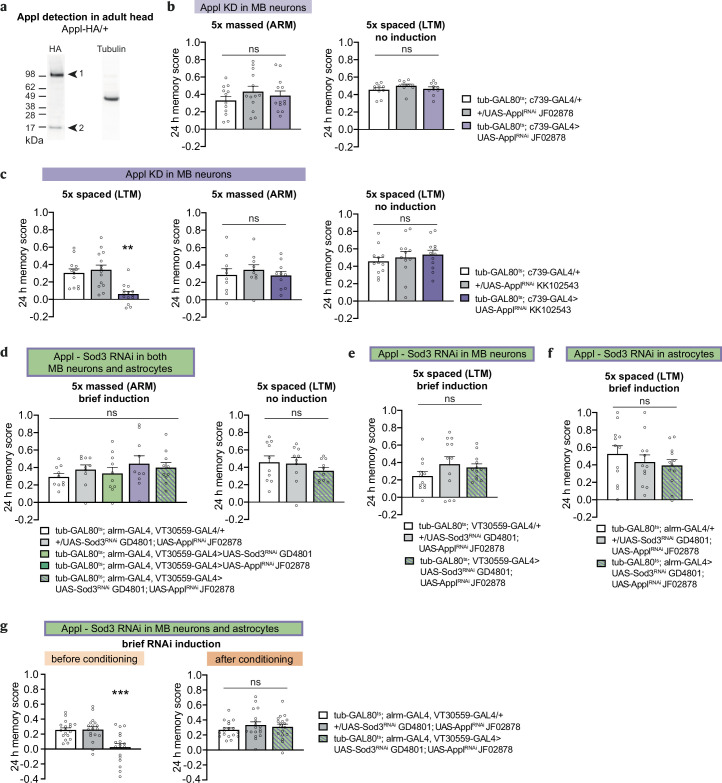
APPL-HA western analysis and additional behavioural experiments, related to Fig. 5. (**a**) Western blot against HA-tagged Appl. The expected size bands are observed at 99 kDa (label 1) and 15 kDa (label 2), corresponding to full-length APPL and the CTF-α fragment, respectively. CTF-α is generated by α-secretase cleavage of full-length Appl. Tubulin was used as a loading control. Twenty replicates of this Western analysis were performed. (**b**) Memory after massed conditioning was not affected by *Appl* KD in adult α/β MB neurons (n = 12 (first group), n = 13 (second and third groups), F_2,35_ = 0.84, P = 0.44). Without *Appl* RNAi induction, memory after spaced training was normal (n = 10, F_2,27_ = 1.19, P = 0.32). (**c**) Memories at 24 h were assessed with a second RNAi against *Appl*. *Appl* KD in adult α/β MB neurons impaired memory after spaced training (n = 12 (first group), n = 14 (second and third groups), F_2,37_ = 11.84, P = 0.0001), but did not affect memory after massed training (n = 10, F_2,27_ = 0.36, P = 0.70). Without *Appl* RNAi induction, memory after spaced training was normal (n = 12, F_2,33_ = 0.52, P = 0.60). (**d**) Mild decreases in both *Appl* and *Sod3* expression in adult MB neurons and astrocytes did not affect memory after massed training (n = 10, F_4,45_ = 0.83, P = 0.515). Without *Appl* and *Sod3* RNAi induction, memory after spaced training was normal (n = 10, F_2,27_ = 0.72, P = 0.50). (**e**) Mild decreases in both *Appl* and *Sod3* expression in adult MB neurons alone did not affect memory after spaced training (n = 12, F_2,33_ = 1.18, P = 0.32). (**f**) Mild decreases in both *Appl* and *Sod3* expression in adult astrocytes alone did not affect memory after spaced training (n = 12, F_2,33_ = 0.70, P = 0.50). (**g**) Brief 1 day induction of both *Appl* and *Sod3* RNAi in astrocytes and MB neurons before spaced conditioning impaired 24 h memory (n = 18, F_2,51_ = 11.88, P = 0.00006), whereas brief 1 day induction of both *Appl* and *Sod3* RNAi after spaced conditioning did not impair 24 h memory (n = 18, F_2,51_ = 0.8017, P = 0.4541). Data are presented as mean ± SEM. Sample sizes for each genotype are listed in the legend in the order of bar appearance. Significance level of the least significant Tukey pairwise comparison following one-way analysis of variance (ANOVA): **P < 0.01; ***P < 0.001; ns: not significant, P > 0.05. Source data

We have shown that H_2_O_2_ is generated extracellularly by Sod3, which requires extracellular Cu^2+^ for its catalytic activity^32^. Because of its potential toxicity, free Cu^2+^ is maintained at a very low concentration in cells and tissues by copper-binding proteins^42^. This raises the question of how sufficient Cu^2+^ levels can be accumulated at the MB synapses to sustain during LTM formation the activity of Sod3 secreted by astrocytes. We hypothesized that Appl, the single fly orthologue of APP transmembrane protein, might provide Cu^2+^ for astrocyte-derived Sod3 activity based on the following observations: (1) the APP extracellular domain has several Cu^2+^ binding sites^43,44^ whose function remains poorly understood^45^, and *Drosophila* Appl carries in its conserved E2 ectodomain the four histidines that are characteristic of the M1 high-affinity Cu^2+^ binding site^46^; (2) APP enables intracellular copper to be transported out of neurons, as APP overexpression decreases Cu^2+^ content in neurons in vitro, whereas *APP* loss of function leads to copper accumulation^47^; (3) *Appl* knockdown in MB neurons induces an LTM defect^48^; and (4) *Appl* exhibits neuronal expression and is enriched in α/β MB neurons^49^, which are involved in LTM^20,36^. We confirmed this strong α/β neuron expression with an HA-tagged *Appl* line generated by CRISPR (Fig. 5a and Extended Data Fig. 7a). Furthermore, we showed that knocking down *Appl* specifically in α/β neurons with RNAi expression induces an LTM defect (Fig. 5b, Extended Data Fig. 7b,c and Supplementary Table 5).

If Appl does provide copper to Sod3, the H_2_O_2_ gradient that forms after spaced conditioning should require Appl. Indeed, we observed that the H_2_O_2_ gradient after spaced conditioning was abolished when Appl expression was inhibited in adult MB (Fig. 5c).

To further support the idea that Appl and Sod3 are involved in the same pathway, we searched for a synergistic interaction between *Appl* and *Sod3* using mild inhibition of their expression. The objective was to demonstrate that the concomitant reduction in *Appl* and *Sod3* expression could result in a more pronounced LTM impairment than that observed in single knockdowns. The flexibility of the GAL4/GAL80^ts^ system, which is induced by elevated temperature, was used to express *Appl* RNAi for a brief period of 1 day, instead of the usual 3 days of RNAi expression. At the behavioural level, the induction of *Appl* or *Sod3* RNAi for a single day in MB neurons and astrocytes did not result in an LTM impairment (Fig. 5d). Next, we asked whether the brief simultaneous induction of *Appl* and *Sod3* RNAi would affect LTM, thus revealing a synergistic interaction. Strikingly, LTM was specifically abolished when both *Appl* and *Sod3* expressions were inhibited in MB neurons and astrocytes for one day (Fig. 5d, Extended Data Fig. 7d and Supplementary Table 5). Of note, the simultaneous mild inhibition of *Appl* and *Sod3* expressions in MB alone or astrocytes alone did not impact LTM (Extended Data Fig. 7e,f). Altogether, we can conclude that the inability to form LTM in double-knockdown flies results from the simultaneous mild decrease of *Appl* expression in the MB, and of *Sod3* expression in astrocytes. To further substantiate this finding, we conducted imaging of H_2_O_2_ dynamics in the double knockdown following brief RNAi expression. Formation of the H_2_O_2_ gradient after spaced conditioning was abolished in flies expressing both *Appl* and *Sod3* RNAi, whereas it remained unaltered in flies expressing a single RNAi, aligning with the behavioural data (Fig. 5e). These results outline the existence of a strong functional interaction between Appl and Sod3, and are in agreement with the hypothesis that Appl supplies copper for Sod3 activity during LTM formation.

To clarify the involvement of the Appl–Sod3 interaction in the early phase of LTM formation rather than in memory recall, which occurs 24 h after conditioning in our experiments, we examined the performance of flies in which the brief *Appl* and *Sod3* RNAi expression was initiated immediately following spaced training, as opposed to before training. The memory performance of flies expressing both *Appl* and *Sod3* RNAi for 1 day after training was not affected (Extended Data Fig. 7g), whereas control flies expressing both RNAi for 1 day before training exhibited an LTM defect (Extended Data Fig. 7g). These results lend support to the hypothesis that Appl–Sod3 interaction is involved in an early step of LTM formation, rather than in LTM recall.

**Fig. 6:**
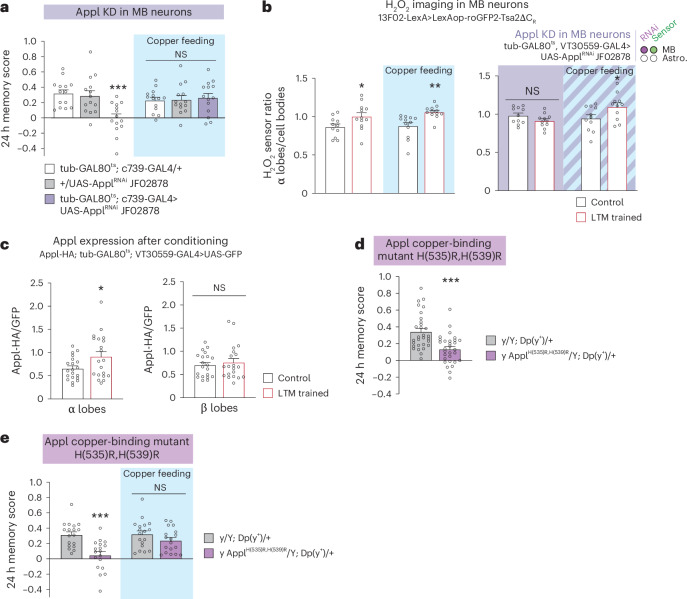
Appl provides copper to sustain Sod3 activity during LTM formation. **a**, Copper feeding (1 mM) 24 h before spaced training restored normal memory capacity in flies with an *Appl* KD in adult α/β MB neurons (two-way ANOVA, *n* = 14, copper treatment: *F*_1,78_ = 0.94, *P* = 0.33; genotype: *F*_2,78_ = 4.48, *P* = 0.01; interaction: *F*_2,78_ = 6.70, *P* = 0.002). **b**, Copper feeding (1 mM) 24 h before training restored the H_2_O_2_ level in α lobes/cell bodies in MB neuron presynaptic terminals of an *Appl* KD in adult MB neurons (right) (two-way ANOVA, *n* = 11, copper treatment: *F*_1,40_ = 3.07, *P* = 0.087; training: *F*_1,40_ = 0.92, *P* = 0.34; interaction: *F*_1,40_ = 7.01, *P* = 0.012). In wild-type controls (left), the increase in H_2_O_2_ level in the α lobes/cell bodies elicited by spaced training was not affected by copper feeding (two-way ANOVA, *n* = 11 (first, third and fourth groups), *n* = 12 (second group), copper treatment: *F*_1,43_ = 0.71, *P* = 0.40; training: *F*_1,43_ = 15.32, *P* = 0,0003; interaction: *F*_1,43_ = 0.30, *P* = 0.59). **c**, Appl expression is increased in MB α lobes after spaced training. Control flies received odorant and shock stimuli temporally dissociated, a condition that does not induce LTM (α lobes: *n* = 20, *t*_38_ = 2.181, *P* = 0.0355; β lobes: *n* = 20, *t*_38_ = 0.5444, *P* = 5894). **d**, Mutations of copper-binding histidines H(535) and H(539) affect memory after spaced training (*n* = 30, *t*_58_ = 4.169, *P* = 0.0001). **e**, Copper feeding (2 mM) 24 h before spaced training restored normal memory capacity in *Appl*^*H(535),H(539)*^ flies (two-way ANOVA, *n* = 18, copper treatment: *F*_1,68_ = 5.603, *P* = 0.0208; genotype: *F*_1,68_ = 17.22, *P* = 0.00009; interaction: *F*_1,68_ = 4.932, *P* = 0.0297). Data are presented as mean ± s.e.m. Genotype sample sizes are listed in the legend in order of bar appearance. Significance level of a two-sided *t*-test or the least significant Tukey pairwise comparison following one-way ANOVA or Šidák pairwise comparison following two-way ANOVA. *P < 0.05; **P < 0.01; ***P < 0.001; NS, not significant, *P* > 0.05. Source data

APP physiological function has been linked to many pathways^50,51^. To further demonstrate that the LTM defect of the *Appl* knockdown is indeed due to a lack of copper, we aimed to rescue the LTM defect of the *Appl* mutant by feeding flies with copper-complemented food. Notably, the memory performance of the *Appl* knockdown was fully rescued by 1 mM copper feeding before conditioning (Fig. 6a). In agreement with this, the H_2_O_2_ gradient was restored after spaced conditioning in the *Appl* knockdown supplemented with copper (Fig. 6b). Thus, copper-supplemented food can compensate for *Appl* knockdown at both the behavioural and H_2_O_2_ signalling levels. Altogether, these results strongly support the idea that the main function of Appl during LTM formation in MB α lobe^20,36^ is to deliver extracellular copper for Sod3.

**Extended Data Fig. 8:**
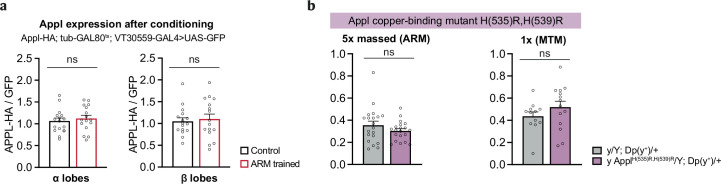
Controls for immunochemical and behavioural experiments, related to Fig. 6. (**a**) No change of Appl expression in MB α and ß lobes after massed training (α lobes: n = 16, t_30_ = 0.5812, P = 0.5655; β lobes: n = 16, t_30_ = 0.3759, P = 0.7096). (**b**) Memory after massed conditioning was not affected in *Appl*^*H(535)R, H(539)R*^ mutant (n = 20, t_38_ = 1.244, P = 0.2209). Memory after 1x conditioning was not affected in *Appl*^*H(535)R, H(539)R*^ mutant (n = 12 (first group), n = 14 (second group), t_24_ = 1.191, P = 0.2452). Data are presented as mean ± SEM. Sample sizes for each genotype are listed in the legend in the order of bar appearance. Significance level of a two-sided t-test: ns: not significant, P > 0.05. Source data

This leads to the question of why the increase in H_2_O_2_ occurs only in the α lobe of the MB, rather than in both the α and β lobes, which are two axonal projections of the same MB neurons. We postulated that this may be due to the activity of Appl itself, given that its copper-delivering property is permissive for H_2_O_2_ production by Sod3. The expression of Appl was monitored in the α and β lobes following spaced conditioning using the *Appl-HA* line and anti-HA immunohistochemistry. The level of Appl-HA expression was normalized to that of green fluorescent protein (GFP), which was expressed homogeneously in MB α/β neurons using the GAL4/UAS system. Notably, Appl-HA expression was specifically elevated in the α lobe following spaced conditioning, whereas no increase was observed in the β lobe (Fig. 6c). Furthermore, no increase in Appl-HA was observed in the α lobe following massed conditioning, a condition that does not engage H_2_O_2_ signalling in α/β neurons (Extended Data Fig. 8a). These findings support the notion that an elevation in Appl-HA levels within the α lobe results in an augmentation of copper release in the surrounding area, thereby facilitating the localized activation of Sod3 secreted by astrocytes.

To further illustrate the pivotal function of the E2 copper-binding domain of Appl in LTM, we generated via CRISPR a constitutive *Appl* mutant for histidines of the evolutionarily conserved E2 copper-binding domain. E2 histidines at positions 535 and 539 were replaced by arginines (Methods). It should be noted that these mutations were introduced into the Appl gene at its typical chromosomal location, without affecting regulatory sequences. *Appl*^*H(535)R,H(539)R*^ mutant with modified copper-binding histidines exhibited an impairment in LTM (Fig. 6d). ARM and middle-term memory remained unaltered in *Appl*^*H(535)R,H(539)R*^ flies (Extended Data Fig. 8b), whereas middle-term memory is affected in *Appl* knockdown^52^. The olfactory acuity and shock reactivity of the *Appl*^*H(535)R,H(539)R*^ mutant were normal (Supplementary Table 6). Notably, the memory defect of *Appl*^*H(535)R,H(539)R*^ mutant was rescued by 24 h 2 mM copper feeding (Fig. 6e). These findings further substantiate the pivotal and specific role of the copper-binding property of Appl in LTM formation.

### Aβ42 inhibits astrocytic nAChRα7 and prevents H_2_O_2_ signalling

**Fig. 7:**
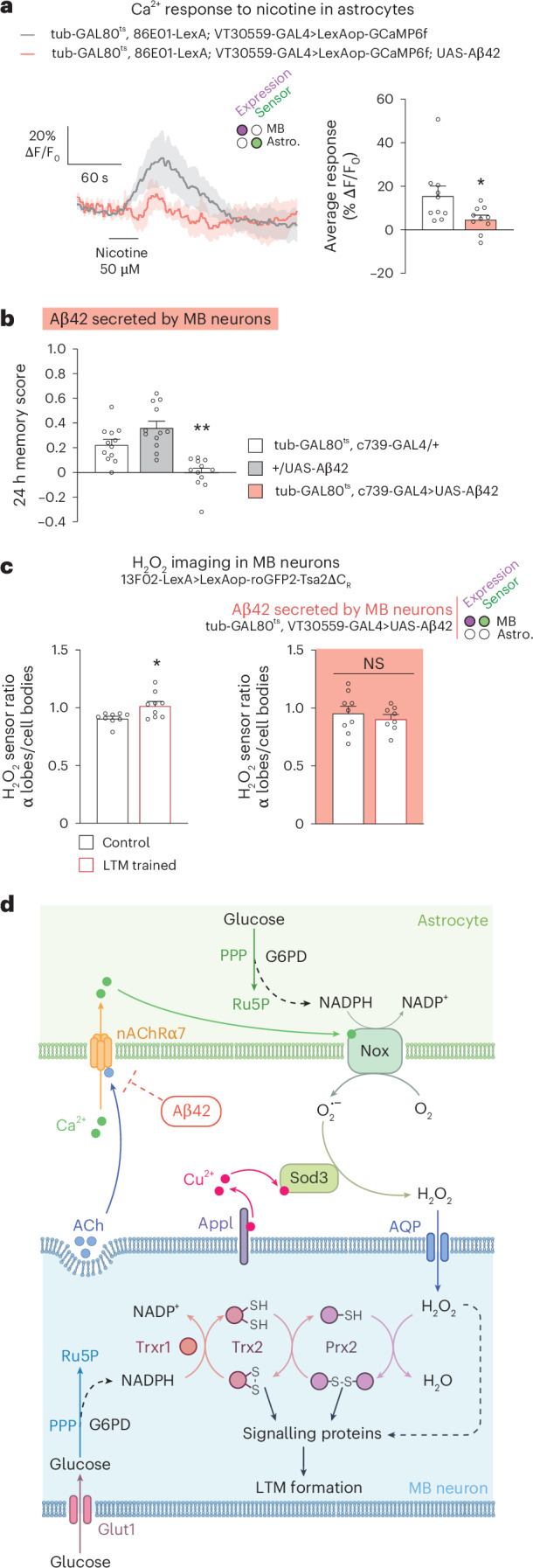
Secreted human Aβ42 inhibits astrocyte-neuron H_2_O_2_ signalling. **a**, Nicotine stimulation (50 µM) elicited increased calcium in astrocytes, which was significantly decreased when secreted human Aβ42 was expressed by MB neurons (*n* = 10, *t*_18_ = 2.29, *P* = 0.03). **b**, Human Aβ42 secretion by adult α/β neurons impaired memory after spaced conditioning (*n* = 12, *F*_2,33_ = 18.51, *P* = 0.000004). **c**, Spaced training elicited an increase in the H_2_O_2_ level of the α lobes/cell bodies as compared with the unpaired control (*n* = 9, *t*_16_ = 2.75, *P* = 0.014), which was impaired by human Aβ42 secretion by adult MB neurons (*n* = 9 (first group), *n* = 8 (second group), *t*_15_ = 0.72, *P* = 0.48). **d**, Model of ANHOS for LTM formation. Ach release from MB neurons^27^ upon spaced training activates astrocytic nicotinic receptor nAChRα7, which induces a calcium elevation in astrocytes, resulting in Nox activation. Nox produces extracellular O_2_•^−^ in the presence of NADPH co-factor derived from astrocytic PPP. In the presence of Cu^2+^ delivered by MB neuronal Appl, extracellular astrocytic Sod3 converts O_2_•^−^ to H_2_O_2_. H_2_O_2_ enters MB neuron lobes through the AQP channel and fuels the redox-sensitive cascade composed of Prx2, Trx2 and Trxr1 enzymes. Regeneration of reduced forms of these enzymes is provided by PPP-derived NADPH produced during LTM formation^17^. Oxidized Prx2 and Trx2 can activate signalling cascades (to be identified), which allows LTM formation. In a parallel pathway, H_2_O_2_ can directly oxidize signalling proteins (dotted line). Aβ42 impedes ANHOS by inhibiting nAChRα7. Data are presented as mean ± s.e.m. Genotype sample sizes are listed in the legend in order of bar appearance. Significance level of a two-sided *t*-test or the least significant Tukey pairwise comparison following one-way ANOVA. *P < 0.05; **P < 0.01; NS, not significant, *P* > 0.05. Source data

**Extended Data Fig. 9:**
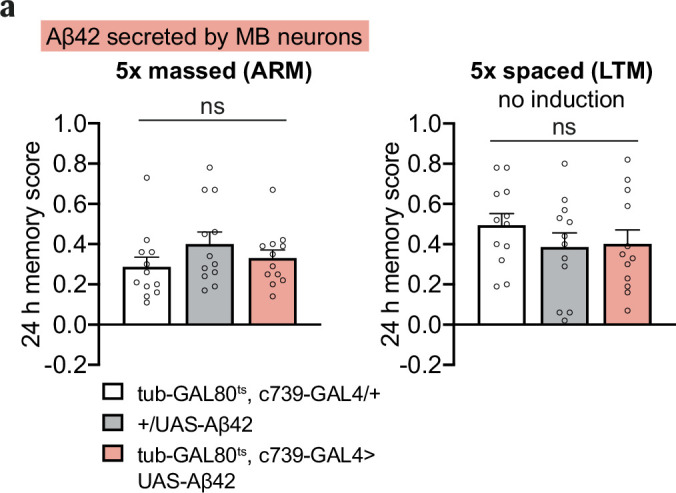
Controls for behavioural experiments, related to Fig. 7. (**a**) Memory after massed conditioning was not affected by secreted human Aß42 secretion by adult MB neurons (n = 12, F_2,33_ = 1.31, P = 0.28). Without Aß42 induction, memory after spaced training was normal (n = 12, F_2,33_ = 0.77, P = 0.47). Data are presented as mean ± SEM. Sample sizes for each genotype are listed in the legend in the order of bar appearance. Significance level of the least significant Tukey pairwise comparison following one-way analysis of variance (ANOVA): ns: not significant, P > 0.05. Source data

Following the observation that Appl is involved in the activation of H_2_O_2_ signalling, we wondered whether, conversely, the toxic derivative of APP, amyloid β (Aβ), can inhibit this signalling pathway. Notably, human Aβ42 is a strong ligand for the nAChRα7 cholinergic receptor^53^. At very low concentrations (<100 pM), which we cannot generate in *Drosophila* using available genetic expression systems, oligomeric Aβ42 was previously shown in vitro to activate nAChRα7 (ref. ^54^); however, in the 10–100 nM range, Aβ42 becomes a strong inhibitor of nAChRα7, preventing activation of the receptor by its natural ligand, ACh^55^. As we showed here that nAChRα7 activation in astrocytes initiates an H_2_O_2_ signalling pathway, we postulated that secreted Aβ might impede this process. Because Appl shows no clear sequence similarity with APP at the level of the Aβ sequence^56^, we expressed a secreted form of the human Aβ42 peptide, as frequently performed in *Drosophila* to study the consequences of brain amyloid expression^57,58^. The human Aβ42 peptide was expressed with a signal sequence that ensures its secretion into the extracellular space^56^. After several days of constitutive human Aβ42 expression in the *Drosophila* brain, diffuse aggregates are observed, along with neurodegenerative defects^57^. These defects are not observed after 3 days of Aβ42 induction^57^. As we aimed to evaluate the potential acute effect of Aβ42 on astrocytic nAChRα7, expression was activated in MB α/β neurons for a short 24-h period. We observed that the calcium response to nicotine was inhibited in the presence of Aβ42 (Fig. 7a), suggesting that human Aβ42 secreted by MB neurons inhibits astrocytic nAChRα7. We then showed that expressing Aβ42 in MB for 24 h affected LTM specifically (Fig. 7b, Extended Data Fig. 9a and Supplementary Table 7). Last, we showed that Aβ42 inhibits formation of the H_2_O_2_ gradient in MB neurons upon spaced conditioning (Fig. 7c). These results demonstrate that an acute expression of toxic Aβ42 in young flies prevents H_2_O_2_ formation by astrocytes after spaced conditioning, and therefore LTM formation.

## Discussion

ROS are byproducts produced mainly by mitochondria, and they are particularly toxic for neurons^2^. It has been suggested that ROS play a negative role in normal cognitive aging^59^, and that a defect in redox homeostasis is involved in the memory impairment that is characteristic of AD^59,60^. Glia help neurons fighting oxidative stress in various ways, including the production of reductive glutathione^10,61,62^ or accumulation of peroxidated lipids transferred from neurons^63^; however, ROS also play physiological roles^1^, and modulating the level of enzymes involved in ROS metabolism affects synaptic plasticity^2^ and learning and memory^8,10^. Nevertheless, the precise mechanisms controlling ROS signalling during memory formation have remained poorly known, in particular because imaging physiological ROS variations during memory formation was out of reach until the development of a genetically encoded highly sensitive sensor^16^. Moreover, it was not known if glia could transfer beneficial ROS to neurons for neuronal plasticity and memory formation. Our data now provide a mechanistic understanding of the role of H_2_O_2_ signalling in vivo during memory formation. We have deciphered a pathway by which astrocytes deliver beneficial H_2_O_2_ to neurons for LTM formation, a phenomenon we have named astrocyte-to-neuron H_2_O_2_ signalling (ANHOS) (Fig. 7d). The production of ROS by astrocytic Nox and Sod3 enzymes, which follows ACh neurotransmitter-induced Ca^2+^ increase, ensures a local H_2_O_2_ accumulation in the MB α lobe, the specific axonal projections encoding LTM^20,36^. Following H_2_O_2_ import, a signalling cascade is initiated by redox-sensitive enzymes^64^ comprising Prx2, Trx2 and Trxr1 (Fig. 7d). We put forth the proposition that this cascade serves as a trigger for LTM formation through the reversible oxidation of specific cysteines in LTM-relevant signalling proteins, in conjunction with the potential direct action of H_2_O_2_ itself. Although the future investigation of these molecular changes will be important, assessing transient changes of oxidation states is technically challenging, and this will require targeting the small subpopulation of α/β neurons that respond to the odorant used during LTM conditioning^65^. What is the potential connection between ANHOS and cAMP-response element binding protein (CREB), a transcription factor that has been demonstrated to play a pivotal role in LTM formation in *Drosophila* and mice^24,25^? Mitogen-activated protein kinases (MAPKs) have been demonstrated to activate CREB by phosphorylation, thereby facilitating memory consolidation^66,67^. Moreover, kinases of the MAPK family, such as p38a and ERK, can be activated by H_2_O_2_ signalling^68,69^. It is therefore proposed that the link between H_2_O_2_ signalling in the α lobe and the regulation of transcription by CREB may imply the activation of the MAPK pathway and retrograde signalling from the axonal projection to the nucleus.

Sod3 secreted by astrocytes plays a central role in ANHOS, as it generates the H_2_O_2_ molecules that will be imported by neurons. Sod3 requires a source of free copper for its catalytic activity. Although the free copper concentration remains extremely low in the brain because of its toxicity, copper concentrations around 100 µM have been reported at the synaptic cleft in mammals^70^. Our results in *Drosophila* suggest that an essential source of copper for the activity of Sod3 secreted by astrocytes comes from neuronal Appl, which exhibits an evolutionarily conserved copper-binding site on its extracellular domain. Indeed, *Appl* and *Sod3* knockdowns display a strong synergistic interaction (Fig. 5d,e) and feeding copper to *Appl* knockdown flies rescued the LTM defect (Fig. 6a) and reestablished the H_2_O_2_ gradient in the olfactory memory centre (Fig. 6b). In addition, mutation of two of four evolutionarily conserved histidines within the E2 copper-binding domain of Appl resulted in impaired LTM (Fig. 6d), whereas middle-term memory remained unaltered (Extended Data Fig. 8b). Conversely, *Appl* knockdown affects both LTM and middle-term memory^52^, which further delineates the specific role of the copper-binding domain in LTM. The study of the *Drosophila* histidine E2 mutant is particularly important because, to the best of our knowledge, no mouse mutants for E2 copper-binding histidines have been reported so far. Feeding copper to *Appl* E2 mutant rescued the LTM defect (Fig. 6e), outlining the essential role of the copper-binding property of Appl in LTM formation.

Altogether, our results suggest that the copper-binding property of Appl and its functional interaction with Sod3 are the major roles of Appl in LTM formation^48^. Of note, expression of extracellular *Sod3* in the human brain is much stronger in astrocytes than in neurons^71^, as in *Drosophila*^33^ (Extended Data Fig. 2c). In mammals, APP binds copper^43,44^; it is expressed at the presynaptic active zone of neurons, where neurotransmitters are released^72,73^; and it is involved in synaptic plasticity and memory^51,74^. We propose that our model developed in *Drosophila* (Fig. 7d) can be generalized to these mammalian data, leading us to conclude that APP activates astrocytic Sod3 for memory formation in mammals. In the mammalian brain, glutamate is the predominant excitatory neurotransmitter. The ANHOS cascade may be activated not only at the cholinergic synapse, but also at the glutamatergic synapse, following calcium signalling in astrocytes that results from the activity of glutamatergic neurons^75^.

From a pathological standpoint, our findings demonstrate that human Aβ42 exerts an inhibitory effect on the ANHOS cascade. This is evidenced by its capacity to impede the Ca^2+^ influx observed in astrocytes following nAChRα7 stimulation, as well as the H_2_O_2_ increase in the MB LTM centre subsequent to spaced conditioning. It is likely that the effect of Aβ42 involves the inhibition of nAChRα7 in astrocytes, which in turn prevents the formation of ROS by astrocytic Nox and Sod3. We propose that this inhibitory process is at play during AD initiation. Indeed, several observations are in agreement with the pathophysiological hypothesis that impairment of astrocytes by diffusible Aβ42 oligomers at cholinergic synapses may occur precociously in the AD brain: (1) soluble synaptic Aβ42 correlates better with the pattern of cognitive decline in AD than amyloid plaques^15^; (2) nAChRα7 is expressed by human brain astrocytes, in particular in the hippocampus^29^, a neural structure that plays a major role in episodic and spatial memory; (3) Aβ42 inhibits nAChRα7 in vitro at a nM concentration^76^; and (4) in humans, pathology of the basal forebrain cholinergic neurons precedes cortical defects but is predictive of future memory deficits^77,78^. In addition, as revealed in a global data-mining survey, *Sod3* has been associated with AD in several ‘omics’ approaches, although its implication remains to be understood^79^. Our ANHOS model integrates observations of early AD and cholinergic neurons, and proposes a key role for *Sod3* in AD. In normal conditions, cholinergic neurons of the basal forebrain, which send diffuse projections to many cortical areas as well as the hippocampus, are involved in memory formation^77,80^. The transition from a physiological condition to an AD condition might therefore involve a switch from an ANHOS activation by ACh to ANHOS inhibition via increased Aβ42 production.

## Methods

### Experimental model

Flies (*Drosophila* *melanogaster*) were raised on standard medium (inactivated yeast 6% *w*/*v*; corn flour 6.7% *w*/*v*; agar 0.9% *w*/*v*; methyl-4-hydroxybenzoate 22 mM), at 18 or 23 °C (depending on the experiments; see respective details below) and 60% humidity in a 12-h light–dark cycle. The study was performed on 1–3-day-old adult flies. For behavioural experiments, both male and female flies were used. For imaging experiments, female flies were used because of their larger size. Before imaging or behavioural experiments, we chose flies informally in a random manner from a much larger group raised together for all studies; there was no formal randomization procedure for selecting flies. Data collection and analysis were not performed blind to the conditions of the experiments. Transgenic flies were outcrossed for five generations to a reference strain carrying the *w*^*1118*^ mutation in an otherwise Canton-Special genetic background. Because TRiP RNAi transgenes are labelled by a *y*^+^ marker, these lines were outcrossed to a *y*^1^ *w*^*67c23*^ strain in an otherwise Canton-Special background. All strains used in this study are described in Supplementary Table 8.

### Behavioural experiments

For behavioural experiments, flies were raised on standard medium at 18 °C and 60% humidity in a 12-h light–dark cycle. We used the GAL4/GAL80^ts^ TARGET system^81^ to inducibly express RNAi constructs exclusively in the MB or astrocytes of adult flies, and not during development. To achieve the induction of RNAi expression, adult flies were kept at 30.5 °C for 3 days before conditioning, unless otherwise specified. To induce Aβ42 carrying the pre-proenkephalin signal peptide for secretion^58^, adult flies were kept at 30.5 °C for 24 h before conditioning. To test the interaction of *Appl* and *Sod3*, adult flies were kept at 30.5 °C for 24 h to achieve mild expression of RNAis before conditioning (Fig. 5d and Extended Data Fig. 7g) or after conditioning (Extended Data Fig. 7g). Otherwise, for non-induced experiments, experimental flies were transferred before conditioning to fresh bottles at 18 °C.

All behavioural experiments, including the sample sizes, were conducted similarly to other studies from our laboratory^17,20,37^. Groups of 20–50 flies were subjected to one of the following olfactory conditioning protocols at 25 °C: a single training cycle (1×), five consecutive associative training cycles (5× massed training), or five associative cycles spaced by 15-min inter-trial intervals (5× spaced training). A non-associative control protocol (unpaired protocol) was also employed for imaging experiments and Appl-HA immunochemistry experiments, during which the odour and shock stimuli were delivered separately in time, with shocks occurring 3 min before the first odorant. Conditioning was performed using previously described barrel-type machines that allow the parallel training of up to six groups^17,20,36^. Throughout the conditioning protocol, each barrel was plugged into a constant air flow at 2 l min^−1^. For a single cycle of associative training, flies were first exposed to an odorant (the CS^+^) for 1 min, while 12 pulses of 5-s long 60-V electric shocks were delivered; flies were then exposed 45 s later to a second odorant without shocks (the CS-) for 1 min. The odorants 3-octanol (Fluka 74878, Sigma-Aldrich) and 4-methylcyclohexanol (Fluka 66360, Sigma-Aldrich), diluted in paraffin oil to a final concentration of 2.79 × 10^−1^ g l^−1^, were alternately used as conditioned stimuli. During unpaired conditionings, the odour and shock stimuli were delivered separately in time, with shocks occurring 3 min before the first odorant.

Flies were kept at 18 °C on standard medium between conditioning and the memory test, except in experiments in which immediate memory was tested after spaced conditioning. The memory test was performed in a T-maze apparatus^82^, 24 h after massed or spaced training, at 25 °C. The memory test was performed at 2 h after 1× training. Each arm of the T-maze was connected to a bottle containing 3-octanol and 4-methylcyclohexanol, diluted in paraffin oil to a final concentration identical to the one used for conditioning. Flies were given 1 min to choose between either arm of the T-maze. A performance score was calculated as the number of flies avoiding the conditioned odour minus the number of flies preferring the conditioned odour, divided by the total number of flies. A single performance index value is the average of two scores obtained from two groups of genotypically identical flies conditioned in two reciprocal experiments, using either odorant (3-octanol or 4-methylcyclohexanol) as the CS^+^. The indicated ‘*n*’ is the number of independent performance index values for each genotype. To avoid giving disproportionate statistical weight to a small number of flies, rare behavioural experiments involving a group of fewer than six flies were excluded.

The shock response tests were performed at 25 °C by placing flies in two connected compartments; electric shocks were provided in only one of the compartments. Flies were given 1 min to move freely in these compartments, after which they were trapped, collected and counted. The compartment where the electric shocks were delivered was alternated between two consecutive groups. Shock avoidance was calculated as for the memory test.

Because the delivery of electric shocks can modify olfactory acuity, our olfactory avoidance tests were performed on flies that had first been presented another odour paired with electric shocks. Innate odour avoidance was measured in a T-maze similar to those used for memory tests, in which one arm of the T-maze was connected to a bottle with odour diluted in paraffin oil and the other arm was connected to a bottle with paraffin oil only. Naive flies were given the choice between the two arms during 1 min. The odour-interlaced side was alternated for successively tested groups. Odour concentrations used in this assay were the same as for the memory assays. At these concentrations, both odorants are innately repulsive.

### In vivo calcium imaging

Calcium imaging experiments were performed on flies expressing the GCaMP6f calcium sensor in astrocytes via the alrm-GAL4 driver, in combination with UAS-GCaMP6f. Transgenes were expressed in astrocytes using the inducible tub-GAL80^ts^; alrm-GAL4 driver. As carried out previously in our laboratory for imaging experiments^17,37^, flies were raised at 23 °C to increase the expression level of genetically encoded sensors without allowing RNAi expression. Adult flies were kept at 30.5 °C for 3 days before conditioning to achieve the induction of RNAi expression.

As in all previous imaging work from our laboratory, all in vivo imaging was performed on female flies, which are preferred as their larger size facilitates surgery. A single fly was picked and prepared for imaging as previously described^17,37^. In brief, the head capsule was opened and the brain was exposed by gently removing the superior tracheae. The head capsule was bathed in artificial haemolymph solution for the duration of the preparation. The composition of this solution was: NaCl 130 mM (Sigma, S9625), KCl 5 mM (Sigma, P3911), MgCl_2_ 2 mM (Sigma, M9272), CaCl_2_ 2 mM (Sigma, C3881), d-trehalose 5 mM (Sigma, 9531), sucrose 30 mM (Sigma, S9378) and HEPES hemisodium salt 5 mM (Sigma, H7637). At the end of surgery, any remaining solution was absorbed and a fresh 100-μl droplet of this solution was applied on top of the brain. Two-photon imaging was performed using a Leica TCS-SP5 upright microscope equipped with a ×25, 0.95 NA water-immersion objective. Two-photon excitation was achieved using a Mai Tai DeepSee laser tuned to 910 nm. The frame rate was one image per second. Recordings were acquired in the astrocytic region in the anterior part of the brain (where MB vertical lobes are located), at approximately 30 µm depth from the top of the brain. Calcium imaging experiments with nicotine stimulation were performed as previously described^17^. Nicotine was freshly diluted from a commercial liquid (Sigma, N3876) into the saline used for imaging on each experimental day. A perfusion setup at a flux of 2.5 ml min^−1^ enabled the time-restricted application of 50 μM nicotine on top of the brain^17^. Baseline recording was performed during 1 min, after which the saline supply was switched to drug supply. The solution reached the in vivo preparation within 30 s. The stimulation was maintained for 30 s, before switching back to the saline perfusion for an additional 5 min. The GCaMP6f intensity was measured in the anterior part of the brain where MB vertical lobes are located. GCaMP6f signal was calculated over time after background subtraction and normalized by a baseline value calculated over the 30 s preceding drug injection using MATLAB software (MathWorks).

### In vivo H_2_O_2_ imaging

H_2_O_2_ imaging experiments were performed on flies expressing the excitation ratiometric H_2_O_2_ sensor roGFP2-Tsa2ΔC_R_^16^ in MB neurons via the 13F02-LexA driver, in combination with LexAop-roGFP2-Tsa2ΔC_R_ (generated in this study). Transgenes were expressed in astrocytes using the inducible tub-GAL80^ts^; alrm-GAL4 driver, or in MB neurons using the inducible tub-GAL80^ts^; VT30559-GAL4 driver. For imaging experiments, flies were raised at 23 °C, except for the imaging experiment after brief RNAi expression for which flies were raised at 18 °C (Fig. 5e). Adult flies were kept at 30.5 °C for 3 days before conditioning to achieve the induction of RNAi expression. For experiments on conditioned flies, data were collected indiscriminately from 30 min to 2 h after training.

Surgery was performed as for calcium imaging. At the end of surgery, any remaining solution was absorbed and a fresh 100-μl droplet of saline solution was applied on top of the brain. Two-photon imaging was performed using a pulsed infra-red laser (Insight X3, Spectra Physics), coupled to a Leica SP8 Dive upright microscope equipped with a ×20, 1.0 NA water-immersion objective and non-descanned spectral hybrid detectors. Emission was collected from 500 to 570 nm.

To characterize the roGFP2-Tsa2ΔC_R_ probe for two-photon excitation microscopy, excitation spectra were measured from 780 to 1,060 nm in steps of 8 nm. Oxidation of the probe was obtained by applying 2 mM H_2_O_2_ (final concentration) on top of the brain. Spectra in the basal and oxidized states were measured before and 15 min after H_2_O_2_ treatment, respectively. Excitation wavelengths of 780 nm and 988 nm were selected for further experiments, as they maximized the sensor emission ratio (ratio 780 nm to 988 nm) between the basal and oxidized states. Experiments after conditioning were performed as follows: on a given brain area, two z-stacks (one at each acquisition wavelength) were consecutively acquired with a step of 1 µm and a line averaging of 3. The detector gain was strictly similar between the two stacks and was set to the 988 nm excitation wavelength at the highest value to avoid any pixel saturation; however, because of differential signal strength within different cellular compartments of MB neurons, two sets of stacks were acquired for each brain: one encompassing the MB lobes, and one covering the soma and calyx area, with different detector gains. For data analysis, the average intensity of three consecutive planes of the stack was calculated for each region of interest at the two excitation wavelengths. Oxidation of the sensor by H_2_O_2_ was measured by the ratio of the signal collected upon 780 nm excitation over the signal collected upon 988 nm excitation (referred to as ‘H_2_O_2_ sensor ratio’), using Leica Microsystems software LAS X Small (offline version).

### Dietary copper supplementation

Copper (II) sulfate pentahydrate (Thermo Fisher Scientific, 197730010) was added to melted standard food medium to achieve a final concentration of 1 mM (Fig. 6a,b), a concentration that does not affect survival rate under chronic exposure^83^. Flies were kept on regular food medium for 48 h at 30.5 °C to achieve RNAi induction and then transferred to copper-enriched medium for 24 h at 30.5 °C for both behavioural and H_2_O_2_ imaging experiments. No lethality was observed with this copper feeding diet. Control flies were transferred on regular medium for 24 h at 30.5 °C.

For the copper rescue of *Appl*^*H(535),H(539)*^ flies (Fig. 6e), copper (II) sulfate pentahydrate was added to melted standard food medium to achieve a final concentration of 2 mM. Flies were kept on copper-enriched medium for 24 h at 18 °C before spaced conditioning.

### Immunohistochemistry

Before dissection, whole adult female flies (2–4 days old) were fixed in 4% paraformaldehyde in PBST (phosphate-buffered saline (PBS) containing 1% Triton X-100) at 4 °C overnight. Brains were dissected in PBS solution and rinsed three times for 20 min in PBST, blocked with 2% bovine serum albumin (Sigma-Aldrich, A9085) in PBST for 2 h at room temperature, and then incubated with primary antibodies at 1:200 dilution (rat anti-HA, Roche, ROAHAHA 11867423001, clone 3F10) in the blocking buffer (2% BSA in PBST) at 4 °C overnight. The following day, brains were rinsed three times for 20 min in PBST, and incubated with secondary antibodies at 1:400 dilution (goat anti-rat Alexa 488, Invitrogen, A11006) in blocking buffer for 3 h at room temperature. Brains were rinsed for a further three times for 20 min in PBST and were mounted in ProLong Mounting Medium (Lifetechnology) for imaging. Images were acquired with a Nikon A1R confocal microscope with a ×20 objective.

In experiments where Appl-HA expression was quantified in MB neurons following spaced conditioning, artificial GFP expression was used as an expression control. *Appl-HA/+; tub-GAL80*^*ts*^*/+; VT30559-GAL4*>*UAS-mCD8GFP* females were raised at 18 °C and transferred for 1.5 days at 30.5 °C to induce GFP expression in MB neurons. The flies were trained using the various protocols. Two hours following conditioning, adult females were fixed in 4% paraformaldehyde in PBST (PBS containing 1% Triton X-100) at 4 °C overnight. For immunochemical analysis, brains were dissected in PBS solution and rinsed three times for 20 min in PBST. They were then blocked with 2% bovine serum albumin (Sigma-Aldrich, A9085) in PBST for 2 h at room temperature. Subsequently, the samples were incubated with primary antibodies at a dilution of 1:200 dilution for rat anti-HA (Roche, ROAHAHA 11867423001, clone 3F10) and 1:400 dilution for mouse anti-GFP (Developmental Studies Hybridoma Bank, DSHB-GFP-12A6, clone 12A6) in blocking buffer (2% BSA in PBST) at 4 °C overnight. On the subsequent day, the brains were rinsed three times for 20 min in PBST and incubated with secondary antibodies at a dilution of 1:400 (goat anti-rat Alexa 594, Invitrogen, A11007 and goat anti-mouse Alexa 488, Invitrogen, A11029) in blocking buffer for 3 h at room temperature, and subsequently placed at 4 °C overnight. Subsequently, the brains were rinsed three times for 20 min in PBST and mounted in ProLong Mounting Medium (Life Technologies) for imaging. Images were obtained using a Nikon A1R confocal microscope with a ×20 objective.

### Western blot

Proteins were extracted from adult female heads after liquid nitrogen snap freezing and mechanical grinding in lysis buffer containing sucrose 100 mM, KH_2_PO_4_/HPO_4_^−^ 40 mM, EDTA 30 mM, KCl 50 mM, 0.25% Triton X-100, dithiothreitol 10 mM, PMSF 0.5 mM and 1× protease inhibitors (Roche, 11836153001). Protein extracts containing loading buffer NOVEX Tricine SDS sample buffer (Thermo Fisher Scientific, LC1676) were run in NOVEX 10–20% Tricine gels (Thermo Fisher, EC66252BOX) and then transferred on nitrocellulose membranes. Membranes were saturated with 15% non-fat milk, PBS–Tween 0.2% for 1 h. Membranes were incubated with primary anti-HA antibodies diluted in PBS–Tween 0.2% blocking medium (mouse anti-HA, 1:5,000 dilution, BioLegend, 901513, clone 16B12) overnight at 4 °C under agitation. Membranes were rinsed five times for 8 min in PBS–Tween 0.2% and incubated with secondary antibodies for 2 h at room temperature under agitation (HRP anti-mouse, 1:10,000 dilution, Promega, W4021). Membranes were further rinsed five times for 8 min in PBS–Tween 0.2%. Revelation was conducted using NOVEX ECL substrate (Thermo Fisher, WP20005) and chemiluminescence was detected with ImageQuant LAS4000. The same procedure was then applied to the same membranes with anti-tubulin antibodies (mouse anti-tubulin, 1:40,000 dilution, Sigma-Aldrich, T16199, clone DM1A). The molecular weight ladder corresponds to Invitrogen SeeBlue Plus2 (LC5925).

### Quantitative PCR

The efficiency of the knockdowns (KDs) used in this study was validated by quantitative PCR with reverse transcription (RT–qPCR) to measure the messenger RNA of the target gene (Supplementary Table 9). Female flies carrying the elav-Gal4 pan-neuronal driver or the repo-Gal4 pan-glial driver were crossed either with males carrying the specified UAS-RNAi or with CS males. Fly progeny were reared at 25 °C throughout their development. Then, 0–1-day-old flies were transferred to fresh food for 1 d before RNA extraction. RNA extraction and complementary DNA synthesis were performed as described elsewhere^68,69^ using the same reagents: the RNeasy Plant Mini kit (QIAGEN), RNA MinElute Cleanup kit (QIAGEN), oligo(dT)20 primers and the SuperScript III First-Strand kit (Life Technologies). The level of complementary DNA for each gene of interest was compared against the level of the α-Tub84B (Tub, CG1913) reference cDNA. Amplification was performed using a LightCycler 480 (Roche) and the SYBR Green I Master mix (Roche). Reactions were carried out in triplicate. The specificity and size of amplification products were assessed by melting curve analyses. Expression relative to the reference was expressed as a ratio (2 − ΔCp, where Cp is the crossing point). The primers used in this study and results of RT–qPCR data are presented in Supplementary Table 9. For KDs that did not yield memory impairments (negative results), both RNAis were checked by qPCR. For KDs that were induced with two distinct RNAis, at least one of the two RNAi lines was validated by qPCR. Exceptions were for those RNAi constructs that were already validated by RT–qPCR in previous studies, in which case the corresponding reference is indicated (Supplementary Table 10).

### Generation of transgenic flies

For the generation of the *LexAop-roGFP2-Tsa2ΔC*_*R*_ *Drosophila* line, the p415 TEF roGFP2-Tsa2ΔCR plasmid (Addgene, #83238)^16^ was digested by NotI and XbaI. The resulting 1,416-bp fragment was purified by electrophoresis and cloned into a pJFRC19 plasmid (13XLexAop2-IVS-myr::GFP, Addgene, #26224)^84^. The resulting construct was verified by sequencing (the molecular cloning was outsourced to RD-Biotech, France). Transgenic fly strains were obtained by site-specific embryonic injection of the resulting vector in the attP18 landing site (X chromosome), which was outsourced to Rainbow Transgenic Flies.

The *Appl-HA* line was generated using the CRISPR approach (outsourced to inDroso). The 3×HA sequence (GCCGCCGTGTACCCCTACGACGTGCCCGACTACGCCGGCTACCCCTACGACGTGCCCGACTACGCCG GCTCCTACCCCTACGACGTGCCCGACTACGCCCCCGCCGCC) preceded by a linker (sequence: GGCGTGGGC) was inserted between the 11th exon and the 3′ UTR of the *Appl* gene using a guide RNA targeting the sequence AAGTGAAA | GAGTAAGCGAGA. The genomic edition was strictly restricted to the 3×HA tag and linker, preventing any alterations due to the presence of a selection marker (scarless).

### Generation of *Appl* copper-binding mutant by CRISPR

The *Appl*^*H(535)R,H(539)R*^ lines was generated using the CRISPR approach, which was outsourced to Rainbow Transgenic Flies. To generate *Appl*^*H(535)R,H(539)R*^, two guide RNAs targeting CCCACGCCTTGGCCCACTAC|CGG and GCGCGCCCTGCACAAGGACC|GGG were employed. The original wild-type genomic sequence GCCCTGCACAAGGACCGGGCCCACGCCTTGGCCCACTACCGGCACCTATTGAACTCTGG, which corresponds to the amino-acids sequence ALHKDRAH(535)ALAH(539)YRHLLNS, was altered to GCCCTGCACAAGGACCGGGCCCGCGCCTTGGCCCGCTACCGGCACCTATTGAACTCTGG, which corresponds to the amino-acids sequence ALHKDRAR(535)ALAR(539)YRHLLNS. The CRISPR strategy was designed to introduce only the specified H/R modifications, and in particular, no marker was associated with the CRISPR-induced mutation. The presence of mutations in *Appl* was confirmed by Rainbow Transgenic Flies through genomic sequencing.

*Appl* mutations were generated on a X chromosome carrying *y w* mutations in an uncontrolled genetic background. To introduce the Canton-Special background appropriate for behavioural experiments, we crossed and *y Appl*^*H(535)R,H(539)R*^ *w* females to wild-type Canton-Special males. At the next F1 generation *y Appl*^*H(535)R,H(539)R*^ *w/+* females were crossed to Canton-Special males. Given the close proximity of the *y* gene to *Appl* and the very low rate of recombination between these two loci (less than 0.03%, as estimated from Flybase data^85^), the presence of *y* mutation was employed to recover *y Appl* mutations in a Canton-Special background. In the F2 generation, recombination between the *y Appl* and *w* loci was screened for, and *y Appl*^*H(535)R,H(539)R*^ *w*^*+*^*/Y* males in a Canton-Special background were recovered. Homozygous *y Appl*^*H(535)R,H(539)R*^ lines was generated. Concurrently, the parental *y w* line, which was utilized to induce the CRISPR mutations, was outcrossed with the identical protocol to generate a control *y* line in a Canton-Special background.

In light of the evidence indicating that *y* mutations impact LTM (based on our own unpublished observations), to conduct behavioural experiments we crossed *y Appl*^*H(535)R,H(539R)*^ females to males carrying the *y+* duplication *PBac{y[+]-attP-3B}VK00033* (ref. ^86^) in a Canton-Special background, designated *Dp(y*^*+*^*)*. The offspring were evaluated in behavioural assays to determine the phenotypes of hemizygous *y Appl*^*H(535)R,H(539)R*^*/Y; Dp(y*^*+*^*)/+* males compared with *y/Y; Dp(y*^*+*^*)/+* males.

### *Sod3* transcript levels

Relative expression of *Sod3* in astrocytes and α/β neurons was obtained from published single-cell transcriptomic data^33^, according to clustering performed in the original study where astrocytes are found in cluster 10 and α/β in cluster 22.

### Quantification and statistical analysis

All data are presented as mean ± s.e.m. For behaviour experiments, two groups of about 30 flies were reciprocally conditioned, using respectively octanol or methylcyclohexanol as the CS^+^. The memory score was calculated from the performance of two groups as described above, which represents a single experimental replicate. For imaging experiments, one replicate corresponds to one fly brain. No statistical methods were used to pre-determine sample sizes but our sample sizes are similar to those reported in previous publications^17,19,37^. Data distribution was assumed to be normal but this was not formally tested. Comparisons of the data series between two conditions were achieved by a two-tailed unpaired *t*-test. Comparisons between more than two distinct groups were made using a one-way analysis of variance (ANOVA) test, followed by Tukey pairwise comparisons between the experimental groups and their controls. ANOVA results are presented as the value of the Fisher distribution F_x,y_ obtained from the data, where *x* is the number of degrees of freedom between groups and *y* is the total number of degrees of freedom for the distribution. For copper-rescue experiments (Fig. 6a,b), two-way ANOVA tests were performed, followed by Šidák’s multiple comparisons test. Statistical tests were performed using the GraphPad Prism 8 software. In the figures, asterisks denote the significance level of the *t*-test, or of the least significant pairwise comparison following ANOVA, with *P < 0.05; **P < 0.01; ***P < 0.001; NS, not significant, *P* > 0.05. Figures were designed using Adobe Illustrator.

### Reporting summary

Further information on research design is available in the Nature Portfolio Reporting Summary linked to this article.

## Supplementary information

Supplementary Information Supplementary Data Tables 1–10.

Reporting Summary

Supplementary Data Statistical Data Source for Supplementary Tables.

## Source data

Source Data Fig. 1 Statistical Source Data for Fig. 1.

Source Data Fig. 2 Statistical Source Data for Fig. 2.

Source Data Fig. 3 Statistical Source Data for Fig. 3.

Source Data Fig. 4 Statistical Source Data for Fig. 4.

Source Data Fig. 5 Statistical Source Data for Fig. 5.

Source Data Fig. 6 Statistical Source Data for Fig. 6.

Source Data Fig. 7 Statistical Source Data for Fig. 7.

Source Data Extended Data Fig. 1 Statistical Source Data for Extended Data Fig. 1.

Source Data Extended Data Fig. 2 Statistical Source Data for Extended Data Fig. 2.

Source Data Extended Data Fig. 3 Statistical Source Data for Extended Data Fig. 3.

Source Data Extended Data Fig. 4 Statistical Source Data for Extended Data Fig. 4.

Source Data Extended Data Fig. 5 Statistical Source Data for Extended Data Fig. 5.

Source Data Extended Data Fig. 6 Statistical Source Data for Extended Data Fig. 6.

Source Data Extended Data Fig. 7 Statistical Source Data for Extended Data Fig. 7.

Source Data Extended Data Fig. 7 Unprocessed gels for Extended Data Fig. 7.

Source Data Extended Data Fig. 8 Statistical Source Data for Extended Data Fig. 8.

Source Data Extended Data Fig. 9 Statistical Source Data for Extended Data Fig. 9.

## Data Availability

No datasets that require mandatory deposition into a public database were generated during the current study. Source data that are reported as graphs on figures and extended data figures are available as Supplementary Information alongside the paper. Additional raw data will be shared with no restriction by the corresponding authors upon request. This study made use of the SCOPE database of single-cell transcriptomics in the fly brain to compare Sod3 expression in neurons and glia (Extended Data Fig. 2c) at https://scope.aertslab.org/#/Davie_et_al_Cell_2018/Davie_et_al_Cell_2018%2FAerts_Fly_AdultBrain_Filtered_57k.loom/gene. Source data are provided with the paper.

## Code availability

This paper does not report original code. Any additional information required to reanalyse the data reported in this paper is available from the corresponding authors upon request.

## References

[1] Sies, H. et al. Defining roles of specific reactive oxygen species (ROS) in cell biology and physiology. *Nat. Rev. Mol. Cell Biol.* **23**, 499–515 (2022). 35190722 10.1038/s41580-022-00456-z

[2] Massaad, C. A. & Klann, E. Reactive oxygen species in the regulation of synaptic plasticity and memory. *Antioxid. Redox Signal.* **14**, 2013–2054 (2011). 20649473 10.1089/ars.2010.3208 PMC3078504

[3] Lambeth, J. D. & Neish, A. S. Nox enzymes and new thinking on reactive oxygen: a double-edged sword revisited. *Annu. Rev. Pathol.* **9**, 119–145 (2014). 24050626 10.1146/annurev-pathol-012513-104651

[4] Klann, E., Roberson, E. D., Knapp, L. T. & Sweatt, J. D. A role for superoxide in protein kinase C activation and induction of long-term potentiation. *J. Biol. Chem.* **273**, 4516–4522 (1998). 9468506 10.1074/jbc.273.8.4516

[5] Kishida, K. T. et al. Synaptic plasticity deficits and mild memory impairments in mouse models of chronic granulomatous disease. *Mol. Cell. Biol.* **26**, 5908–5920 (2006). 16847341 10.1128/MCB.00269-06 PMC1592752

[6] Oswald, M. C. et al. Reactive oxygen species regulate activity-dependent neuronal plasticity in *Drosophila*. *eLife* **7**, e39393 (2018). 30540251 10.7554/eLife.39393 PMC6307858

[7] Dhawan, S. et al. Reactive oxygen species mediate activity-regulated dendritic plasticity through NADPH oxidase and aquaporin regulation. *Front. Cell. Neurosci.* **15**, 641802 (2021). 34290589 10.3389/fncel.2021.641802 PMC8288108

[8] Levin, E. D. et al. Molecular manipulations of extracellular superoxide dismutase: functional importance for learning. *Behav. Genet.* **28**, 381–390 (1998). 9926619 10.1023/a:1021673703129

[9] Santello, M., Toni, N. & Volterra, A. Astrocyte function from information processing to cognition and cognitive impairment. *Nat. Neurosci.* **22**, 154–166 (2019). 30664773 10.1038/s41593-018-0325-8

[10] Vicente-Gutierrez, C. et al. Astrocytic mitochondrial ROS modulate brain metabolism and mouse behaviour. *Nat. Metab.* **1**, 201–211 (2019). 32694785 10.1038/s42255-018-0031-6

[11] Butterfield, D. A. & Halliwell, B. Oxidative stress, dysfunctional glucose metabolism and Alzheimer disease. *Nat. Rev. Neurosci.* **20**, 148–160 (2019). 30737462 10.1038/s41583-019-0132-6 PMC9382875

[12] Tönnies, E. & Trushina, E. Oxidative stress, synaptic dysfunction, and Alzheimer’s disease. *J. Alzheimers Dis. JAD* **57**, 1105–1121 (2017). 28059794 10.3233/JAD-161088 PMC5409043

[13] Musiek, E. S. & Holtzman, D. M. Three dimensions of the amyloid hypothesis: time, space and ‘wingmen’. *Nat. Neurosci.* **18**, 800–806 (2015). 26007213 10.1038/nn.4018 PMC4445458

[14] Padmanabhan, P., Kneynsberg, A. & Götz, J. Super-resolution microscopy: a closer look at synaptic dysfunction in Alzheimer disease. *Nat. Rev. Neurosci.* **22**, 723–740 (2021). 34725519 10.1038/s41583-021-00531-y

[15] Palop, J. J. & Mucke, L. Amyloid-β-induced neuronal dysfunction in Alzheimer’s disease: from synapses toward neural networks. *Nat. Neurosci.* **13**, 812–818 (2010). 20581818 10.1038/nn.2583 PMC3072750

[16] Morgan, B. et al. Real-time monitoring of basal H_2_O_2_ levels with peroxiredoxin-based probes. *Nat. Chem. Biol.* **12**, 437–443 (2016). 27089028 10.1038/nchembio.2067

[17] de Tredern, E. et al. Glial glucose fuels the neuronal pentose phosphate pathway for long-term memory. *Cell Rep.* **36**, 109620 (2021). 34433052 10.1016/j.celrep.2021.109620 PMC8411112

[18] Silva, B. et al. Glia fuel neurons with locally synthesized ketone bodies to sustain memory under starvation. *Nat. Metab.* **4**, 213–224 (2022). 35177854 10.1038/s42255-022-00528-6 PMC8885408

[19] Rabah, Y. et al. Glycolysis-derived alanine from glia fuels neuronal mitochondria for memory in *Drosophila*. *Nat. Metab.* **5**, 2002–2019 (2023). 37932430 10.1038/s42255-023-00910-y PMC10663161

[20] Pascual, A. & Préat, T. Localization of long-term memory within the *Drosophila* mushroom body. *Science* **294**, 1115–1117 (2001). 11691997 10.1126/science.1064200

[21] Tully, T., Preat, T., Boynton, S. C. & Del Vecchio, M. Genetic dissection of consolidated memory in *Drosophila*. *Cell* **79**, 35–47 (1994). 7923375 10.1016/0092-8674(94)90398-0

[22] Kawahara, T., Quinn, M. T. & Lambeth, J. D. Molecular evolution of the reactive oxygen-generating NADPH oxidase (Nox/Duox) family of enzymes. *BMC Evol. Biol.* **7**, 109 (2007). 17612411 10.1186/1471-2148-7-109 PMC1940245

[23] Ritsick, D. R., Edens, W. A., Finnerty, V. & Lambeth, J. D. Nox regulation of smooth muscle contraction. *Free Radic. Biol. Med.* **43**, 31–38 (2007). 17561091 10.1016/j.freeradbiomed.2007.03.006 PMC1989158

[24] Widmer, Y. F. et al. Multiple neurons encode CrebB dependent appetitive long-term memory in the mushroom body circuit. *eLife* **7**, e39196 (2018). 30346271 10.7554/eLife.39196 PMC6234028

[25] Hirano, Y. et al. Shifting transcriptional machinery is required for long-term memory maintenance and modification in *Drosophila* mushroom bodies. *Nat. Commun.* **7**, 13471 (2016). 27841260 10.1038/ncomms13471 PMC5114576

[26] Stincone, A. et al. The return of metabolism: biochemistry and physiology of the pentose phosphate pathway. *Biol. Rev. Camb. Philos. Soc.* **90**, 927–963 (2015). 25243985 10.1111/brv.12140 PMC4470864

[27] Barnstedt, O. et al. Memory-relevant mushroom body output synapses are cholinergic. *Neuron* **89**, 1237–1247 (2016). 26948892 10.1016/j.neuron.2016.02.015 PMC4819445

[28] Séguéla, P., Wadiche, J., Dineley-Miller, K., Dani, J. A. & Patrick, J. W. Molecular cloning, functional properties, and distribution of rat brain alpha 7: a nicotinic cation channel highly permeable to calcium. *J. Neurosci.* **13**, 596–604 (1993). 7678857 10.1523/JNEUROSCI.13-02-00596.1993 PMC6576637

[29] Yu, W.-F., Guan, Z.-Z., Bogdanovic, N. & Nordberg, A. High selective expression of α7 nicotinic receptors on astrocytes in the brains of patients with sporadic Alzheimer’s disease and patients carrying Swedish APP 670/671 mutation: a possible association with neuritic plaques. *Exp. Neurol.* **192**, 215–225 (2005). 15698636 10.1016/j.expneurol.2004.12.015

[30] Zhang, K. et al. Fear learning induces α7-nicotinic acetylcholine receptor-mediated astrocytic responsiveness that is required for memory persistence. *Nat. Neurosci.* **24**, 1686–1698 (2021). 34782794 10.1038/s41593-021-00949-8

[31] Schieber, M. & Chandel, N. S. ROS function in redox signaling and oxidative stress. *Curr. Biol.* **24**, R453–R462 (2014). 24845678 10.1016/j.cub.2014.03.034 PMC4055301

[32] Wang, Y., Branicky, R., Noë, A. & Hekimi, S. Superoxide dismutases: dual roles in controlling ROS damage and regulating ROS signaling. *J. Cell Biol.* **217**, 1915–1928 (2018). 29669742 10.1083/jcb.201708007 PMC5987716

[33] Davie, K. et al. A single-cell transcriptome atlas of the aging *Drosophila* brain. *Cell* **174**, 982–998.e20 (2018). 29909982 10.1016/j.cell.2018.05.057 PMC6086935

[34] Perea, G., Navarrete, M. & Araque, A. Tripartite synapses: astrocytes process and control synaptic information. *Trends Neurosci.* **32**, 421–431 (2009). 19615761 10.1016/j.tins.2009.05.001

[35] Kremer, M. C., Jung, C., Batelli, S., Rubin, G. M. & Gaul, U. The glia of the adult *Drosophila* nervous system. *Glia* **65**, 606–638 (2017). 28133822 10.1002/glia.23115 PMC5324652

[36] Yu, D., Akalal, D.-B. G. & Davis, R. L. *Drosophila* α/βmushroom body neurons form a branch-specific, long-term cellular memory trace after spaced olfactory conditioning. *Neuron* **52**, 845–855 (2006). 17145505 10.1016/j.neuron.2006.10.030 PMC1779901

[37] Plaçais, P.-Y. et al. Upregulated energy metabolism in the *Drosophila* mushroom body is the trigger for long-term memory. *Nat. Commun.* **8**, 15510 (2017). 28580949 10.1038/ncomms15510 PMC5465319

[38] Bienert, G. P. & Chaumont, F. Aquaporin-facilitated transmembrane diffusion of hydrogen peroxide. *Biochim. Biophys. Acta* **1840**, 1596–1604 (2014). 24060746 10.1016/j.bbagen.2013.09.017

[39] Stöcker, S., Van Laer, K., Mijuskovic, A. & Dick, T. P. The conundrum of hydrogen peroxide signaling and the emerging role of peroxiredoxins as redox relay hubs. *Antioxid. Redox Signal.* **28**, 558–573 (2018). 28587525 10.1089/ars.2017.7162

[40] Rhee, S. G. & Kil, I. S. Multiple functions and regulation of mammalian peroxiredoxins. *Annu. Rev. Biochem.* **86**, 749–775 (2017). 28226215 10.1146/annurev-biochem-060815-014431

[41] Bouzaiane, E., Trannoy, S., Scheunemann, L., Plaçais, P.-Y. & Preat, T. Two independent mushroom body output circuits retrieve the six discrete components of *Drosophila* aversive memory. *Cell Rep.* **11**, 1280–1292 (2015). 25981036 10.1016/j.celrep.2015.04.044

[42] D’Ambrosi, N. & Rossi, L. Copper at synapse: release, binding and modulation of neurotransmission. *Neurochem. Int.* **90**, 36–45 (2015). 26187063 10.1016/j.neuint.2015.07.006

[43] Wild, K., August, A., Pietrzik, C. U. & Kins, S. Structure and synaptic function of metal binding to the amyloid precursor protein and its proteolytic fragments. *Front. Mol. Neurosci.* **10**, 21 (2017). 28197076 10.3389/fnmol.2017.00021 PMC5281630

[44] Young, T. R., Pukala, T. L., Cappai, R., Wedd, A. G. & Xiao, Z. The human amyloid precursor protein binds copper ions dominated by a picomolar-affinity site in the helix-rich E2 domain. *Biochemistry* **57**, 4165–4176 (2018). 29894164 10.1021/acs.biochem.8b00572

[45] Cappai, R. et al. The amyloid precursor protein (APP) of Alzheimer disease and its paralog, APLP2, modulate the Cu/Zn-nitric oxide-catalyzed degradation of glypican-1 heparan sulfate in vivo. *J. Biol. Chem.* **280**, 13913–13920 (2005). 15677459 10.1074/jbc.M409179200

[46] Dahms, S. O. et al. Metal binding dictates conformation and function of the amyloid precursor protein (APP) E2 domain. *J. Mol. Biol.* **416**, 438–452 (2012). 22245578 10.1016/j.jmb.2011.12.057

[47] Eskici, G. & Axelsen, P. H. Copper and oxidative stress in the pathogenesis of Alzheimer’s disease. *Biochemistry* **51**, 6289–6311 (2012). 22708607 10.1021/bi3006169

[48] Goguel, V. et al. *Drosophila* amyloid precursor protein-like is required for long-term memory. *J. Neurosci.* **31**, 1032–1037 (2011). 21248128 10.1523/JNEUROSCI.2896-10.2011 PMC6632943

[49] Leyssen, M. et al. Amyloid precursor protein promotes post-developmental neurite arborization in the *Drosophila* brain. *EMBO J.* **24**, 2944–2955 (2005). 16052209 10.1038/sj.emboj.7600757 PMC1187942

[50] Müller, U. C. & Zheng, H. Physiological functions of APP family proteins. *Cold Spring Harb. Perspect. Med.* **2**, a006288 (2012). 22355794 10.1101/cshperspect.a006288 PMC3281588

[51] Ludewig, S. & Korte, M. Novel insights into the physiological function of the APP (gene) family and its proteolytic fragments in synaptic plasticity. *Front. Mol. Neurosci.* **9**, 161 (2016). 28163673 10.3389/fnmol.2016.00161 PMC5247455

[52] Bourdet, I., Preat, T. & Goguel, V. The full-length form of the *Drosophila* amyloid precursor protein is involved in memory formation. *J. Neurosci.* **35**, 1043–1051 (2015). 25609621 10.1523/JNEUROSCI.2093-14.2015 PMC6605540

[53] Wang, H. Y., Lee, D. H., Davis, C. B. & Shank, R. P. Amyloid peptide Aβ(1-42) binds selectively and with picomolar affinity to α7 nicotinic acetylcholine receptors. *J. Neurochem.* **75**, 1155–1161 (2000). 10936198 10.1046/j.1471-4159.2000.0751155.x

[54] Dineley, K. T., Bell, K. A., Bui, D. & Sweatt, J. D. β-Amyloid peptide activates α7 nicotinic acetylcholine receptors expressed in *Xenopus* oocytes. *J. Biol. Chem.* **277**, 25056–25061 (2002). 11983690 10.1074/jbc.M200066200

[55] Liu, Q., Kawai, H. & Berg, D. K. β-Amyloid peptide blocks the response of α7-containing nicotinic receptors on hippocampal neurons. *Proc. Natl Acad. Sci. USA* **98**, 4734–4739 (2001). 11274373 10.1073/pnas.081553598 PMC31903

[56] Guo, Q., Wang, Z., Li, H., Wiese, M. & Zheng, H. APP physiological and pathophysiological functions: insights from animal models. *Cell Res.* **22**, 78–89 (2012). 21769132 10.1038/cr.2011.116 PMC3351924

[57] Iijima, K. et al. Dissecting the pathological effects of human Aβ40 and Aβ42 in *Drosophila*: a potential model for Alzheimer’s disease. *Proc. Natl Acad. Sci. USA* **101**, 6623–6628 (2004). 15069204 10.1073/pnas.0400895101 PMC404095

[58] Finelli, A., Kelkar, A., Song, H.-J., Yang, H. & Konsolaki, M. A model for studying Alzheimer’s Aβ42-induced toxicity in *Drosophila melanogaster*. *Mol. Cell. Neurosci.* **26**, 365–375 (2004). 15234342 10.1016/j.mcn.2004.03.001

[59] Ionescu-Tucker, A. & Cotman, C. W. Emerging roles of oxidative stress in brain aging and Alzheimer’s disease. *Neurobiol. Aging* **107**, 86–95 (2021). 34416493 10.1016/j.neurobiolaging.2021.07.014

[60] Shi, Q. & Gibson, G. E. Oxidative stress and transcriptional regulation in Alzheimer disease. *Alzheimer Dis. Assoc. Disord.* **21**, 276–291 (2007). 18090434 10.1097/WAD.0b013e31815721c3 PMC3109432

[61] Shih, A. Y. et al. Coordinate regulation of glutathione biosynthesis and release by Nrf2-expressing glia potently protects neurons from oxidative stress. *J. Neurosci.* **23**, 3394–3406 (2003). 12716947 10.1523/JNEUROSCI.23-08-03394.2003 PMC6742304

[62] Jimenez-Blasco, D., Santofimia-Castaño, P., Gonzalez, A., Almeida, A. & Bolaños, J. P. Astrocyte NMDA receptors’ activity sustains neuronal survival through a Cdk5–Nrf2 pathway. *Cell Death Differ.* **22**, 1877–1889 (2015). 25909891 10.1038/cdd.2015.49 PMC4648333

[63] Liu, L., MacKenzie, K. R., Putluri, N., Maletić-Savatić, M. & Bellen, H. J. The glia-neuron lactate shuttle and elevated ROS promote lipid synthesis in neurons and lipid droplet accumulation in glia via APOE/D. *Cell Metab.* **26**, 719–737.e6 (2017). 28965825 10.1016/j.cmet.2017.08.024 PMC5677551

[64] Mishina, N. M. et al. Which antioxidant system shapes intracellular H_2_O_2_ gradients? *Antioxid. Redox Signal.* **31**, 664–670 (2019). 30864831 10.1089/ars.2018.7697 PMC6657290

[65] Turner, G. C., Bazhenov, M. & Laurent, G. Olfactory representations by *Drosophila* mushroom body neurons. *J. Neurophysiol.* **99**, 734–746 (2008). 18094099 10.1152/jn.01283.2007

[66] Bozon, B. et al. MAPK, CREB and *zif268* are all required for the consolidation of recognition memory. *Philos. Trans. R. Soc. Lond. B Biol. Sci.* **358**, 805–814 (2003). 12740127 10.1098/rstb.2002.1224 PMC1693143

[67] Medina, J. H. & Viola, H. ERK1/2: a key cellular component for the formation, retrieval, reconsolidation and persistence of memory. *Front. Mol. Neurosci.* **11**, 361 (2018). 30344477 10.3389/fnmol.2018.00361 PMC6182090

[68] Guyton, K. Z., Liu, Y., Gorospe, M., Xu, Q. & Holbrook, N. J. Activation of mitogen-activated protein kinase by H_2_O_2_. *J. Biol. Chem.* **271**, 4138–4142 (1996). 8626753 10.1074/jbc.271.8.4138

[69] Barata, A. G. & Dick, T. P. A role for peroxiredoxins in H_2_O_2_- and MEKK-dependent activation of the p38 signaling pathway. *Redox Biol.* **28**, 101340 (2020). 31629169 10.1016/j.redox.2019.101340 PMC6807362

[70] Gaier, E. D., Eipper, B. A. & Mains, R. E. Copper signaling in the mammalian nervous system: synaptic effects. *J. Neurosci. Res.* **91**, 2–19 (2013). 23115049 10.1002/jnr.23143 PMC3926505

[71] Zhang, Y. et al. Purification and characterization of progenitor and mature human astrocytes reveals transcriptional and functional differences with mouse. *Neuron* **89**, 37–53 (2016). 26687838 10.1016/j.neuron.2015.11.013 PMC4707064

[72] Laßek, M. et al. Amyloid precursor proteins are constituents of the presynaptic active zone. *J. Neurochem.* **127**, 48–56 (2013). 23815291 10.1111/jnc.12358

[73] Guénette, S., Strecker, P. & Kins, S. APP protein family signaling at the synapse: insights from intracellular APP-binding proteins. *Front. Mol. Neurosci.* **10**, 87 (2017). 28424586 10.3389/fnmol.2017.00087 PMC5371672

[74] Lee, S. H. et al. APP family regulates neuronal excitability and synaptic plasticity but not neuronal survival. *Neuron* **108**, 676–690.e8 (2020). 32891188 10.1016/j.neuron.2020.08.011 PMC7704911

[75] Goenaga, J., Araque, A., Kofuji, P. & Herrera Moro Chao, D. Calcium signaling in astrocytes and gliotransmitter release. *Front. Synaptic Neurosci.* **15**, 1138577 (2023). 36937570 10.3389/fnsyn.2023.1138577 PMC10017551

[76] Pettit, D. L., Shao, Z. & Yakel, J. L. β-Amyloid(1-42) peptide directly modulates nicotinic receptors in the rat hippocampal slice. *J. Neurosci.* **21**, RC120 (2001). 11150356 10.1523/JNEUROSCI.21-01-j0003.2001 PMC6762461

[77] Ballinger, E. C., Ananth, M., Talmage, D. A. & Role, L. W. Basal forebrain cholinergic circuits and signaling in cognition and cognitive decline. *Neuron* **91**, 1199–1218 (2016). 27657448 10.1016/j.neuron.2016.09.006 PMC5036520

[78] Schmitz, T. W., Nathan Spreng, R. & Alzheimer’s Disease Neuroimaging Initiative. Basal forebrain degeneration precedes and predicts the cortical spread of Alzheimer’s pathology. *Nat. Commun.* **7**, 13249 (2016). 27811848 10.1038/ncomms13249 PMC5097157

[79] Zhang, M. et al. Drug repositioning for Alzheimer’s disease based on systematic ‘omics’ data mining. *PLoS ONE* **11**, e0168812 (2016). 28005991 10.1371/journal.pone.0168812 PMC5179106

[80] Ananth, M. R., Rajebhosale, P., Kim, R., Talmage, D. A. & Role, L. W. Basal forebrain cholinergic signalling: development, connectivity and roles in cognition. *Nat. Rev. Neurosci.* **24**, 233–251 (2023). 36823458 10.1038/s41583-023-00677-x PMC10439770

[81] McGuire, S. E., Le, P. T., Osborn, A. J., Matsumoto, K. & Davis, R. L. Spatiotemporal rescue of memory dysfunction in *Drosophila*. *Science* **302**, 1765–1768 (2003). 14657498 10.1126/science.1089035

[82] Tully, T. & Quinn, W. G. Classical conditioning and retention in normal and mutant *Drosophila melanogaster*. *J. Comp. Physiol. [A]* **157**, 263–277 (1985). 10.1007/BF01350033 3939242

[83] Bonilla-Ramirez, L., Jimenez-Del-Rio, M. & Velez-Pardo, C. Acute and chronic metal exposure impairs locomotion activity in *Drosophila melanogaster*: a model to study Parkinsonism. *Biometals* **24**, 1045–1057 (2011). 21594680 10.1007/s10534-011-9463-0

[84] Pfeiffer, B. D. et al. Refinement of tools for targeted gene expression in *Drosophila*. *Genetics* **186**, 735–755 (2010). 20697123 10.1534/genetics.110.119917 PMC2942869

[85] Öztürk-Çolak, A. et al. FlyBase: updates to the *Drosophila* genes and genomes database. *Genetics* **227**, 211 (2024). 10.1093/genetics/iyad211 PMC11075543 38301657

[86] Venken, K. J. T., He, Y., Hoskins, R. A. & Bellen, H. J. P[acman]: a BAC transgenic platform for targeted insertion of large DNA fragments in *D. melanogaster*. *Science* **314**, 1747–1751 (2006). 17138868 10.1126/science.1134426

