# Supplementary material for: Astrocyte-to-neuron H2O2 signalling supports long-term memory formation in Drosophila and is impaired in an Alzheimer’s disease model
Source: Nat Metab. 2025 Jan 24;7(2):321–35. doi: 10.1038/s42255-024-01189-3 (PMC11860231; doi:10.1038/s42255-024-01189-3)
Supplement: Supplementary file 1 — Supplementary Data Tables 1–10. [file 42255_2024_1189_MOESM1_ESM.pdf]

nature metabolism

Supplementary information

https://doi.org/10.1038/s42255-024-01189-3

Astrocyte-to-neuron H_2O_2 signalling supports long-term memory formation in *Drosophila* and is impaired in an Alzheimer's disease model

In the format provided by the authors and unedited

Supplementary Tables

Supplementary Data Table 1. Sensory acuity controls related to Fig. 1 and Extended Data fig. 1 and 2.

Genotypes	Shock reactivity		Olfactory acuity			
			Octanol		Methylcyclohexanol	
	Mean ± SEM	Statistics	Mean ± SEM	Statistics	Mean ± SEM	Statistics
tub-GAL80^{ts}; alrm-GAL4/+	0.430 ± 0.058	n = 8<br>$F_{2,21}$ = 2.46<br>P = 0.11	0.589 ± 0.049	n = 10<br>$F_{2,27}$ = 0.15<br>P = 0.87	0.684 ± 0.062	n = 10<br>$F_{2,27}$ = 1.07<br>P = 0.36
+/UAS-Nox RNAi HMS00429	0.430 ± 0.066		0.596 ± 0.069		0.784 ± 0.041	
tub-GAL80^{ts}; alrm-GAL4> UAS- Nox RNAi HMS00429	0.606 ± 0.070		0.637 ± 0.082		0.672 ± 0.072	
tub-GAL80^{ts}; alrm-GAL4/+	0.493 ± 0.062	n = 12<br>$F_{2,33}$ = 0.72<br>P = 0.49	0.449 ± 0.029	n = 12<br>$F_{2,33}$ = 0.015<br>P = 0.98	0.438 ± 0.021	n =12<br>$F_{2,33}$ = 0.030<br>P = 0.97
+/UAS-Nox RNAi KK111991	0.437 ± 0.045		0.449 ± 0.028		0.441 ± 0.038	
tub-GAL80^{ts}; alrm-GAL4> UAS-Nox RNAi KK111991	0.534 ± 0.064		0.455 ± 0.026		0.448 ± 0.029	
tub-GAL80^{ts}; alrm-GAL4/+	0.386 ± 0.053	n = 8<br>$F_{2,21}$ = 1.98<br>P = 0.16	0.739 ± 0.042	n = 10<br>$F_{2,27}$ = 0.20<br>P = 0.82	0.769 ± 0.066	n = 10<br>$F_{2,27}$ = 0.76<br>P = 0.48
+/UAS-G6PD RNAi KK108898	0.311 ± 0.056		0.716 ± 0.053		0.679 ± 0.043	
tub-GAL80^{ts}; alrm-GAL4> UAS-G6PD RNAi KK108898	0.483 ± 0.072		0.684 ± 0.082		0.676 ± 0.070	

tub-GAL80^{ts}; alrm-GAL4/+	0.399 ± 0.066	n = 8<br>$F_{2,21}$ = 1.62<br>P = 0.22	0.609 ± 0.074	n = 10<br>$F_{2,27}$ = 0.87<br>P = 0.43	0.767 ± 0.080	n = 10<br>$F_{2,27}$ = 0.11<br>P = 0.89
+/UAS-Pgd RNAi HMC05959	0.305 ± 0.064		0.732 ± 0.062		0.771 ± 0.039	
tub-GAL80^{ts}; alrm-GAL4> UAS-Pgd RNAi HMC05959	0.456 ± 0.047		0.652 ± 0.065		0.726 ± 0.092	
tub-GAL80^{ts}; alrm-GAL4/+	0.63 ± 0.044	n = 12<br>$F_{2,33}$ = 1.01<br>P = 0.37	0.723 ± 0.034	n = 12<br>$F_{2,33}$ = 0.006<br>P >0.99	0.792 ± 0.038	n = 12<br>$F_{2,33}$ = 0.155<br>P = 0.86
+/UAS-Pgls RNAi HMS02626	0.59 ± 0.052		0.728 ± 0.035		0.783 ± 0.028	
tub-GAL80^{ts}; alrm-GAL4> Pgls RNAi HMS02626	0.69 ± 0.045		0.723 ± 0.032		0.809 ± 0.024	
tub-GAL80^{ts}; alrm-GAL4/+	0.679 ± 0.047	n = 10<br>$F_{2,27}$ = 0.77<br>P = 0.47	0.674 ± 0.036	n = 10<br>$F_{2,27}$ = 1.64<br>P = 0.21	0.801 ± 0.062	n = 10<br>$F_{2,27}$ = 3.16<br>P = 0.06
+/UAS-nAChRα7 RNAi JF02570	0.572 ± 0.069		0.603 ± 0.037		0.613 ± 0.050	
tub-GAL80^{ts}; alrm-GAL4> UAS-nAChRα7 RNAi JF02570	0.623 ± 0.065		0.582 ± 0.040		0.641 ± 0.058	
tub-GAL80^{ts}; alrm-GAL4/+	0.409 ± 0.049	n = 12<br>$F_{2,33}$ = 0.29<br>P = 0.75	0.495 ± 0.019	n = 12<br>$F_{2,33}$ = 0.51<br>P = 0.60	0.535 ± 0.050	n = 12<br>$F_{2,33}$ = 0.18<br>P = 0.84
+/UAS-nAChRα7 RNAi KK108471	0.447 ± 0.054		0.543 ± 0.030		0.507 ± 0.033	
tub-GAL80^{ts}; alrm-GAL4>	0.464 ± 0.053		0.514 ± 0.047		0.500 ± 0.047	

UAS-nAChRα7 RNAi KK108471						
tub-GAL80^{ts}; alrm-GAL4/+	0.431 ± 0.083	n = 8<br>$F_{2,21}$ = 0.30<br>P = 0.75	0.669 ± 0.068	n = 10<br>$F_{2,27}$ = 0.20<br>P = 0.82	0.779 ± 0.066	n = 10<br>$F_{2,27}$ = 0.63<br>P = 0.54
+/UAS-Sod3 RNAi GD4801	0.438 ± 0.062		0.669 ± 0.056		0.673 ± 0.073	
tub-GAL80^{ts}; alrm-GAL4> UAS-Sod3 RNAi GD4801	0.500 ± 0.064		0.620 ± 0.064		0.748 ± 0.067	
tub-GAL80^{ts}; alrm-GAL4/+	0.590 ± 0.054	n = 12<br>$F_{2,33}$ = 0.41<br>P = 0.67	0.662 ± 0.028	n = 12<br>$F_{2,33}$ = 1.60<br>P = 0.22	0.797 ± 0.037	n = 12<br>$F_{2,33}$ = 0.77<br>P = 0.47
+/UAS-Sod3 RNAi GD3774	0.578 ± 0.074		0.738 ± 0.033		0.828 ± 0.023	
tub-GAL80^{ts}; alrm-GAL4> UAS-Sod3 RNAi GD3774	0.654 ± 0.063		0.693 ± 0.030		0.768 ± 0.039	

Supplementary Data Table 2. Sensory acuity controls related to Fig. 3 and Extended Data fig. 3.

<table>
<tr><th rowspan="3">Genotypes</th><th colspan="2" rowspan="2">Shock reactivity</th><th colspan="4">Olfactory acuity</th></tr>
<tr><th colspan="2">Octanol</th><th colspan="2">Methylcyclohexanol</th></tr>
<tr><th>Mean ± SEM</th><th>Statistics</th><th>Mean ± SEM</th><th>Statistics</th><th>Mean ± SEM</th><th>Statistics</th></tr>
<tr><td>tub-GAL80^{ts}; alrm-GAL4/+</td><td>0.595 ± 0.090</td><td rowspan="3">n = 10
$F_{2,27}$ = 0.38
P = 0.69</td><td>0.780 ± 0.036</td><td rowspan="3">n = 10
$F_{2,27}$ = 0.24
P = 0.79</td><td>0.592 ± 0.041</td><td rowspan="3">n = 10
$F_{2,27}$ = 0.84
P = 0.44</td></tr>
<tr><td>+/UAS-human-secreted-catalase</td><td>0.548 ± 0.063</td><td>0.746 ± 0.041</td><td>0.650 ± 0.029</td></tr>
<tr><td>tub-GAL80^{ts}; alrm-GAL4> UAS-human-secreted-catalase</td><td>0.508 ± 0.055</td><td>0.772 ± 0.030</td><td>0.600 ± 0.031</td></tr>
</table>

Supplementary Data Table 3. Sensory acuity controls related to Fig. 4 and Extended Data fig. 4.

<table>
<tr><th rowspan="3">Genotypes</th><th colspan="2" rowspan="2">Shock reactivity</th><th colspan="4">Olfactory acuity</th></tr>
<tr><th colspan="2">Octanol</th><th colspan="2">Methylcyclohexanol</th></tr>
<tr><th>Mean ± SEM</th><th>Statistics</th><th>Mean ± SEM</th><th>Statistics</th><th>Mean ± SEM</th><th>Statistics</th></tr>
<tr><td>tub-GAL80^{ts}; c739-GAL4/+</td><td>0.703 ± 0.057</td><td rowspan="3">n = 12
$F_{2,33}$ = 2.49
P = 0.10</td><td>0.485 ± 0.047</td><td rowspan="3">n = 10
$F_{2,27}$ = 0.31
P = 0.73</td><td>0.503 ± 0.061</td><td rowspan="3">n = 10
$F_{2,27}$ = 0.06
P = 0.94</td></tr>
<tr><td>+/UAS-AQP RNAi HMC03266</td><td>0.546 ± 0.079</td><td>0.465 ± 0.050</td><td>0.523 ± 0.069</td></tr>
<tr><td>tub-GAL80^{ts}; c739-GAL4> UAS-AQP RNAi HMC03266</td><td>0.746 ± 0.062</td><td>0.520 ± 0.052</td><td>0.534 ± 0.058</td></tr>
<tr><td>tub-GAL80^{ts}; c739-GAL4/+</td><td>0.660 ± 0.055</td><td rowspan="3">n = 12
$F_{2,33}$ = 0.17
P = 0.85</td><td>0.597 ± 0.054</td><td rowspan="3">n = 10
$F_{2,27}$ = 0.41
P = 0.67</td><td>0.692 ± 0.060</td><td rowspan="3">n = 10
$F_{2,27}$ = 0.48
P = 0.62</td></tr>
<tr><td>+/UAS-AQP RNAi KK102737</td><td>0.678 ± 0.071</td><td>0.552 ± 0.054</td><td>0.630 ± 0.037</td></tr>
<tr><td>tub-GAL80^{ts}; c739-GAL4> UAS-AQP RNAi KK102737</td><td>0.710 ± 0.060</td><td>0.534 ± 0.043</td><td>0.665 ± 0.032</td></tr>
</table>

Supplementary Data Table 4. Sensory acuity controls related to Fig. 4 and Extended Data fig. 5 and 6.

Genotypes	Shock reactivity		Olfactory acuity			
			Octanol		Methylcyclohexanol	
	Mean ± SEM	Statistics	Mean ± SEM	Statistics	Mean ± SEM	Statistics
tub-GAL80^{ts}; c739-GAL4/+	0.569 ± 0.045	n = 12<br>$F_{2,33}$ = 1.91<br>P = 0.16	0.667 ± 0.058	n = 10<br>$F_{2,27}$ = 0.42<br>P = 0.66	0.627 ± 0.054	n = 10<br>$F_{2,27}$ = 2.71<br>P = 0.08
+/UAS-Prx2 RNAi HMS00935	0.689 ± 0.049		0.643 ± 0.040		0.784 ± 0.039	
tub-GAL80^{ts}; c739-GAL4> UAS-Prx2 RNAi HMS00935	0.628 ± 0.035		0.605 ± 0.044		0.697 ± 0.049	
tub-GAL80^{ts}; c739-GAL4/+	0.573 ± 0.044	n = 12<br>$F_{2,33}$ = 0.42<br>P = 0.66	0.519 ± 0.027	n = 10<br>$F_{2,27}$ = 0.52<br>P = 0.60	0.473 ± 0.029	n = 10<br>$F_{2,27}$ = 1.60<br>P = 0.22
+/UAS-Prx2 RNAi VSH330046	0.558 ± 0.028		0.535 ± 0.021		0.489 ± 0.020	
tub-GAL80^{ts}; c739-GAL4> UAS-Prx2 RNAi VSH330046	0.606 ± 0.039		0.501 ± 0.022		0.535 ± 0.026	
tub-GAL80^{ts}; c739-GAL4/+	0.448 ± 0.059	n = 12<br>$F_{2,33}$ = 0.82<br>P = 0.45	0.548 ± 0.041	n = 12<br>$F_{2,33}$ = 3.43<br>P = 0.04	0.679 ± 0.060	n = 12<br>$F_{2,33}$ = 1.22<br>P = 0.31
+/UAS-Trx2 RNAi HMS00989	0.544 ± 0.077		0.673 ± 0.040		0.713 ± 0.041	
tub-GAL80^{ts}; c739-GAL4> UAS-Trx2 RNAi HMS00989	0.548 ± 0.048		0.560 ± 0.029		0.603 ± 0.050	
tub-GAL80^{ts}; c739-GAL4/+	0.577 ± 0.039	n = 12<br>$F_{2,33}$ = 0.21	0.635 ± 0.046	n = 12<br>$F_{2,33}$ = 0.11	0.664 ± 0.062	n = 12<br>$F_{2,33}$ = 1.12

+/UAS-Trx2 RNAi HMS00603	0.623 ± 0.065	P = 0.81	0.644 ± 0.047	P = 0.89	0.748 ± 0.050	P = 0.34
tub-GAL80^{ts}; c739-GAL4> UAS-Trx2 RNAi HMS00603	0.576 ± 0.070		0.616 ± 0.035		0.643 ± 0.044	
tub-GAL80^{ts}; c739-GAL4/+	0.478 ± 0.077	n = 10 $F_{2,27}$ = 0.52 P = 0.60	0.714 ± 0.044	n = 10 $F_{2,27}$ = 0.90 P = 0.42	0.700 ± 0.038	n = 10 $F_{2,27}$ = 1.47 P = 0.25
+/UAS-Trxr1 RNAi HMS00784	0.561 ± 0.059		0.686 ± 0.036		0.748 ± 0.037	
tub-GAL80^{ts}; c739-GAL4> UAS-Trxr1 RNAi HMS00784	0.467 ± 0.077		0.776 ± 0.062		0.653 ± 0.042	
tub-GAL80^{ts}; c739-GAL4/+	0.485 ± 0.049	n = 12 $F_{2,33}$ = 0.13 P = 0.88	0.674 ± 0.060	n = 10 $F_{2,27}$ = 0.08 P = 0.92	0.606 ± 0.036	n = 10 $F_{2,27}$ = 0.34 P = 0.71
+/UAS-Trxr1 RNAi GD9738	0.518 ± 0.041		0.661 ± 0.058		0.667 ± 0.060	
tub-GAL80^{ts}; c739-GAL4> UAS-Trxr1 RNAi GD9738	0.498 ± 0.048		0.642 ± 0.049		0.650 ± 0.062	

Supplementary Data Table 5. Sensory acuity controls related to Fig. 5 and Extended Data fig. 7.

Genotypes	Shock reactivity		Olfactory acuity			
			Octanol		Methylcyclohexanol	
	Mean ± SEM	Statistics	Mean ± SEM	Statistics	Mean ± SEM	Statistics
tub-GAL80^{ts}; c739-GAL4/+	0.531 ± 0.055	n = 14<br>$F_{2,39}$ = 1.36<br>P = 0.27	0.743 ± 0.058	n = 10<br>$F_{2,27}$ = 0.12<br>P = 0.88	0.635 ± 0.061	n = 10<br>$F_{2,27}$ = 3.84<br>P = 0.03
+/UAS-Appl RNAi JF02878	0.640 ± 0.048		0.730 ± 0.020		0.730 ± 0.062	
tub-GAL80^{ts}; c739-GAL4> UAS- Appl RNAi JF02878	0.612 ± 0.041		0.710 ± 0.055		0.837 ± 0.022	
tub-GAL80^{ts}; c739-GAL4/+	0.640 ± 0.077	n = 12<br>$F_{2,33}$ = 0.61<br>P = 0.55	0.599 ± 0.053	n = 10<br>$F_{2,27}$ = 1.19<br>P = 0.32	0.820 ± 0.034	n = 10<br>$F_{2,27}$ = 0.49<br>P = 0.62
+/UAS-Appl RNAi KK102543	0.529 ± 0.073		0.580 ± 0.067		0.787 ± 0.032	
tub-GAL80^{ts}; c739-GAL4> UAS- Appl RNAi KK102543	0.572 ± 0.065		0.489 ± 0.037		0.762 ± 0.547	
tub-GAL80^{ts}; alrm-GAL4, VT30559-GAL4/+	0.592 ± 0.031	n = 10<br>$F_{2,27}$ = 0.75<br>P = 0.48	0.520 ± 0.055	n = 10<br>$F_{2,27}$ = 3.14<br>P = 0.06	0.810 ± 0.052	n = 10<br>$F_{2,27}$ = 1.03<br>P =0.37
+/ UAS-Sod3 RNAi GD4801; UAS-Appl RNAi KK102543	0.575 ± 0.049		0.660 ± 0.047		0.840 ± 0.033	
tub-GAL80^{ts}; alrm-GAL4, VT30559-GAL4> UAS-Sod3 RNAi GD4801; UAS-Appl RNAi KK102543	0.663 ± 0.073		0.510 ± 0.040		0.760 ± 0.034	

Supplementary Data Table 6. Sensory acuity controls related to Fig. 6 and Extended Data fig. 8.

<table>
<tr><th rowspan="3">Genotypes</th><th colspan="2" rowspan="2">Shock reactivity</th><th colspan="4">Olfactory acuity</th></tr>
<tr><th colspan="2">Octanol</th><th colspan="2">Methylcyclohexanol</th></tr>
<tr><th>Mean ± SEM</th><th>Statistics</th><th>Mean ± SEM</th><th>Statistics</th><th>Mean ± SEM</th><th>Statistics</th></tr>
<tr><td>y/Y; Dp(y⁺)/+</td><td>0.733 ± 0.069</td><td rowspan="2">n = 12
t_{22} = 0.96
P = 0.35</td><td>0.715 ± 0.044</td><td rowspan="2">n = 12
t_{22} = 0.26
P = 0.80</td><td>0.561 ± 0.037</td><td rowspan="2">n = 12
t_{22} = 1.11
P = 0.28</td></tr>
<tr><td>y Appl^{H(535)R,H(539)R}/Y ; Dp(y⁺)/+</td><td>0.635 ± 0.075</td><td>0.733 ± 0.051</td><td>0.631 ± 0.051</td></tr>
</table>

Supplementary data Table 7. Sensory acuity controls related to Fig. 7 and Extended Data fig. 9.

Genotypes	Shock reactivity		Olfactory acuity			
			Octanol		Methylcyclohexanol	
	Mean ± SEM	Statistics	Mean ± SEM	Statistics	Mean ± SEM	Statistics
tub-GAL80^{ts}; c739-GAL4/+	0.550 ± 0.081	n = 10<br>$F_{2,27}$ = 0.95<br>P = 0.40	0.720 ± 0.047	n = 12<br>$F_{2,33}$ = 2.30<br>P = 0.12	0.720 ± 0.052	n = 12<br>$F_{2,33}$ = 1.63<br>P = 0.21
+/UAS-Aβ42	0.410 ± 0.054		0.690 ± 0.048		0.590 ± 0.052	
tub-GAL80^{ts}; c739-GAL4> UAS-Aβ42	0.480 ± 0.067		0.570 ± 0.059		0.620 ± 0.061	

Supplementary Table 8: ***Drosophila melanogaster*** **strains used in the study**

Strain	Source / reference	Main expression (driver)
tub-GAL80^{ts}; alrm-GAL4	de Tredern et al. 2021	astrocytes
tub-GAL80^{ts}; c739-GAL4	Turrel et al. 2018	α/β MB neurons
tub-GAL80^{ts}, 13F02-LexA; VT30559-GAL4	de Tredern et al. 2021	MB neurons (GAL4 and LexA)
tub-GAL80^{ts}, 13F02-LexA; alrm-GAL4	This study	MB neurons (LexA) and astrocytes (GAL4)
tub-GAL80^{ts}; alrm-GAL4, VT30559-GAL4	This study	MB neurons (GAL4) and astrocytes (LexA)
86E01-LexA	Bloomington Drosophila Stock Center (BDSC) 54287	astrocytes
tub-GAL80^{ts}, 86E01-LexA; VT30559-GAL4	This study	MB neurons (GAL4) and astrocytes (LexA)
UAS-Nox^{RNAi} HMS00429	BDSC 32433	N/A
UAS-Nox^{RNAi} KK111991	Vienna Drosophila Resource Center (VDRC) 102559	N/A
UAS-G6PD^{RNAi} KK108898	VDRC 101507	N/A
UAS-Pgd^{RNAi} HMC05959	BDSC 65078	N/A
UAS-Pgls^{RNAi} HMS02626	BDSC 42933	N/A
UAS-nAChRa7^{RNAi} JF02570	BDSC 27251	N/A
UAS-nAChRa7^{RNAi} KK108471	VDRC 100756	N/A
UAS-Sod3^{RNAi} GD4801	VDRC 37793	N/A
UAS-Sod3^{RNAi} GD3774	VDRC 8760	N/A
UAS-Glut1^{RNAi} KK108683	VDRC 101365	N/A
UAS-AQP^{RNAi} HMC03266	BDSC 51504	N/A
UAS-AQP^{RNAi} KK102737	VDRC 109314	N/A
UAS-Prx2^{RNAi} HMS00935	BDSC 34971	N/A
UAS-Prx2^{RNAi} VSH330046	VDRC 330046	N/A
UAS-Trx2^{RNAi} HMS00989	BDSC 34019	N/A
UAS-Trx2^{RNAi} HMS00603	BDSC 33721	N/A

UAS-Trxr1^{RNAi} HMS00784	BDSC 32984	N/A
UAS-Trxr1^{RNAi} GD9738	VDRC 47308	N/A
UAS-Appl^{RNAi} JF02878	BDSC 28043	N/A
UAS-Appl^{RNAi} KK102543	VDRC 108312	N/A
UAS-Aß42	Finelli et al. 2004	N/A
UAS-GCaMP6f	BDSC 42747	N/A
LexAop-GCaMP6f	BDSC 44277	N/A
LexAop-GCaMP6f; UAS-Aß42	This study	N/A
UAS-GCaMP6f; UAS-nAChRa7^{RNAi} JF02570	This study	N/A
LexAop-roGFP-Tsa2DC$_R$	This study	N/A
LexAop-roGFP-Tsa2DC$_R$; UAS-Glut1^{RNAi} KK108683	This study	N/A
LexAop-roGFP-Tsa2DC$_R$; UAS-Nox^{RNAi} HMS00429	This study	N/A
LexAop-roGFP-Tsa2DC$_R$; UAS-G6PD^{RNAi} KK108898	This study	N/A
LexAop-roGFP-Tsa2DC$_R$; UAS-nAChRa7^{RNAi} JF02570	This study	N/A
LexAop-roGFP-Tsa2DC$_R$; UAS-Sod3^{RNAi} GD4801	This study	N/A
LexAop-roGFP-Tsa2DC$_R$; UAS-AQP^{RNAi} HMC03266	This study	N/A
LexAop-roGFP-Tsa2DC$_R$; UAS-Appl^{RNAi} JF02878	This study	N/A
LexAop-roGFP-Tsa2DC$_R$; UAS-Aß42	This study	N/A
Appl-HA	This study	N/A
Appl-HA; tub-GAL80^{ts}; VT30559-GAL4	This study	MB neurons
y Appl$^{H(535)R,H(539)R}$	This study	N/A
PBac{y[+]-attP-3B}VK00033	BDSC 9750	N/A
UAS-human-secreted-catalase	Dhawan et al. 2021	N/A
UAS-Bib^{RNAi} GD1049	VDRC 8893	N/A

UAS-Bib^{RNAi} *JF02771*	BDSC 27691	N/A
UAS-Drip^{RNAi} *GD1936*	VDRC 51936	N/A
UAS-Drip^{RNAi} *HMC0945*	BDSC 44661	N/A
UAS-Prip^{RNAi} *GD3499*	VDRC 8124	N/A
UAS-Prip^{RNAi} *HMC03097*	BDSC 50695	N/A

Supplementary Table 9: RNAi efficiency measured by RT-qPCR and primers' sequences used for mRNA expression assays

RNAi tested relative to control Repo-GAL4/+	Forward sequence	Reverse sequence	RNAi efficiency (% mean of mRNA levels ± SEM)	Statistics
Repo-GAL4/UAS-Sod3^{RNAi} GD3774	GTTAGCCTGGCACTCTGTG	GGGTCCAATCAGATAGGCAATG	-46.1 ± 7.4	n = 4<br>$t_6 = 3.387$<br>$P = 0.0147$
Repo-GAL4/UAS-AQP^{RNAi} HMC03266 and KK102737	ACCCATCCTGATCAACGAG	GTACATGGACACGCCAAAG	HMC03266:<br>-93.2 ± 2.5<br>KK102737:<br>-96.3 ± 0.5	n = 3-4<br>$F_{2,8} = 21.39$<br>$P = 0.0006$
Repo-GAL4/UAS-Prx2^{RNAi} HMS00935	AAGGAAGCAGGGTGGTCTG	GATGCCAGTCTCCTCATCGAG	-71.8 ± 8.0	n = 4<br>$t_6 = 5.703$<br>$P = 0.0013$
Repo-GAL4/UAS-Prx2^{RNAi} VSH330046			-6.7 ± 16.0	n = 3-4<br>$t_5 = 0.3138$<br>$P = 0.7663$
Repo-GAL4/UAS-Trx2^{RNAi} HMS00989	TCGATGTGGACGAATGCGAA	GACCTTGACGCCGTTCTTGA	-82.3 ± 2.4	n = 4<br>$t_6 = 17.06$<br>$P < 0.0001$
Repo-GAL4/UAS-Trx2^{RNAi} HMS00603			-79.4 ± 4.6	n = 3-4<br>$t_5 = 8.321$<br>$P = 0.0004$
Repo-GAL4/UAS-Trxr1^{RNAi} HMS00784	TGGATCTGCGCGACAAGAAAG	GAAGGTCTGGGCGGTGATTG	-65.3 ± 5.1	n = 4<br>$t_6 = 5.997$<br>$P = 0.001$
Repo-GAL4/UAS-Bib^{RNAi} GD8893	GTAGTGCGATCCATATCTCCGA	GGCACAGTTACGCCATAAAGC	-34.5 ± 4.9	n = 4<br>$t_6 = 4.094$<br>$P = 0.0064$

Repo-GAL4/UAS-Drip^{RNAi} GD1936	CAAATCGCATTCACCTTTGGC	CACCAACGATCAGGAATCCAAG	-53.1 ± 9.5	n = 8<br>$t_{14} = 4.64$<br>P = 0.0004
Repo-GAL4/UAS-Prip^{RNAi} GD3499	CTGCCGTTACCGCTGGAAT	CAAGCACTGGAAAACCACATAGA	-9.8 ± 0.8	n = 3-4<br>$t_5 = 3.139$<br>P = 0.0257

Supplementary Table 10: RNAi used in this study and previously validated in the literature

<table>
<tr><th>RNAi</th><th>Reference</th></tr>
<tr><td>Nox HMS00429</td><td>(1)</td></tr>
<tr><td>Nox KK111991</td><td>(2)</td></tr>
<tr><td>G6PD KK108898</td><td rowspan="4">(3)</td></tr>
<tr><td>Pgd HMC05959</td></tr>
<tr><td>Pgls HMS02626</td></tr>
<tr><td>nAChRα7 JF02570 and KK108471</td></tr>
<tr><td>Sod3 GD4801</td><td>(4)</td></tr>
<tr><td>Glut1 KK108683</td><td>(3)</td></tr>
<tr><td>Bib JF02771</td><td rowspan="2">(5)</td></tr>
<tr><td>Drip HMC02945</td></tr>
<tr><td>Prip HMC03097</td><td>(6)</td></tr>
<tr><td>Appl JF02878</td><td>(7)</td></tr>
<tr><td>Appl KK102543</td><td>(8)</td></tr>
</table>

1. S. Lang, T. A. Hilsabeck, K. A. Wilson, A. Sharma, N. Bose, D. J. Brackman, J. N. Beck, L. Chen, M. A. Watson, D. W. Killilea, S. Ho, A. Kahn, K. Giacomini, M. L. Stoller, T. Chi, P. Kapahi, A conserved role of the insulin-like signaling pathway in diet-dependent uric acid pathologies in Drosophila melanogaster. *PLOS Genet.* **15**, e1008318 (2019).

2. F. J.-M. Chartier, É. J.-L. Hardy, P. Laprise, Crumbs limits oxidase-dependent signaling to maintain epithelial integrity and prevent photoreceptor cell death. *J. Cell Biol.* **198**, 991–998 (2012).

3. E. De Tredern, Y. Rabah, L. Pasquer, J. Minatchy, P.-Y. Plaçais, T. Preat, Glial glucose fuels the neuronal pentose phosphate pathway for long-term memory. *Cell Rep.* **36**, 109620 (2021).

4. I. Jung, T.-Y. Kim, J. Kim-Ha, Identification of *Drosophila* SOD3 and its protective role against phototoxic damage to cells. *FEBS Lett.* **585**, 1973–1978 (2011).

5. D. Sobrido-Cameán, M. C. W. Oswald, D. M. D. Bailey, A. Mukherjee, M. Landgraf, Activity-regulated growth of motoneurons at the neuromuscular junction is mediated by NADPH oxidases. *Front. Cell. Neurosci.* **16**, 1106593 (2023).

6. A. H. York-Andersen, B. W. Wood, E. L. Wilby, A. S. Berry, T. T. Weil, Osmolarity-regulated swelling initiates egg activation in *Drosophila*. *Open Biol.* **11**, 210067 (2021).

7. C. Song, K. Broadie, Fragile X mental retardation protein coordinates neuron-to-glia communication for clearance of developmentally transient brain neurons. *Proc. Natl. Acad. Sci.* **120**, e2216887120 (2023).

8. I. A. Kessissoglou, D. Langui, A. Hasan, M. Maral, S. B. Dutta, P. R. Hiesinger, B. A. Hassan, The Drosophila amyloid precursor protein homologue mediates neuronal survival and neuroglial interactions. *PLOS Biol.* **18**, e3000703 (2020).
