# Supplementary material for: Astrocyte-to-neuron H2O2 signalling supports long-term memory formation in Drosophila and is impaired in an Alzheimer’s disease model
Source: Nat Metab. 2025 Jan 24;7(2):321–35. doi: 10.1038/s42255-024-01189-3 (PMC11860231; doi:10.1038/s42255-024-01189-3)
Supplement: Supplementary file 2 — Reporting Summary [file 42255_2024_1189_MOESM2_ESM.pdf]

nature portfolio

Corresponding author(s): Thomas PREAT
Last updated by author(s): Nov 15th, 2024

Reporting Summary

Nature Portfolio wishes to improve the reproducibility of the work that we publish. This form provides structure for consistency and transparency in reporting. For further information on Nature Portfolio policies, see our Editorial Policies and the Editorial Policy Checklist.

Statistics

For all statistical analyses, confirm that the following items are present in the figure legend, table legend, main text, or Methods section.

n/a	Confirmed	
☐	☒	The exact sample size (n) for each experimental group/condition, given as a discrete number and unit of measurement
☐	☒	A statement on whether measurements were taken from distinct samples or whether the same sample was measured repeatedly
☐	☒	The statistical test(s) used AND whether they are one- or two-sided<br>*Only common tests should be described solely by name; describe more complex techniques in the Methods section.*
☒	☐	A description of all covariates tested
☐	☒	A description of any assumptions or corrections, such as tests of normality and adjustment for multiple comparisons
☐	☒	A full description of the statistical parameters including central tendency (e.g. means) or other basic estimates (e.g. regression coefficient) AND variation (e.g. standard deviation) or associated estimates of uncertainty (e.g. confidence intervals)
☐	☒	For null hypothesis testing, the test statistic (e.g. F, t, r) with confidence intervals, effect sizes, degrees of freedom and P value noted<br>*Give P values as exact values whenever suitable.*
☒	☐	For Bayesian analysis, information on the choice of priors and Markov chain Monte Carlo settings
☒	☐	For hierarchical and complex designs, identification of the appropriate level for tests and full reporting of outcomes
☒	☐	Estimates of effect sizes (e.g. Cohen's d, Pearson's r), indicating how they were calculated

Our web collection on statistics for biologists contains articles on many of the points above.

Software and code

Policy information about availability of computer code

Data collection	In vivo imaging data were obtained on two distinct commercial Leica microscopes and acquired through the Leica proprietary softwares.
Data analysis	- Statistical analysis was done using GraphPad Prism 8 (GraphPad Software).<br>- H2O2 and calcium in vivo imaging data were analyzed manually (ROI tracing and intensity calculation) using LAS-X (3.5.7.23225) and MATLAB (9.4.0.813654) softwares.<br>- Figures were designed on Adobe illustrator 2022.

For manuscripts utilizing custom algorithms or software that are central to the research but not yet described in published literature, software must be made available to editors and reviewers. We strongly encourage code deposition in a community repository (e.g. GitHub). See the Nature Portfolio guidelines for submitting code & software for further information.

Data

Policy information about availability of data

All manuscripts must include a data availability statement. This statement should provide the following information, where applicable:

- Accession codes, unique identifiers, or web links for publicly available datasets
- A description of any restrictions on data availability
- For clinical datasets or third party data, please ensure that the statement adheres to our policy

No datasets that require mandatory deposition into a public database were generated during the current study. Source data that are reported as graphs on figures

and extended data figures are available as Supplementary Information alongside the paper. Additional raw data will be shared with no restriction by the corresponding authors upon request.
This study made use of the SCOPE database of single-cell transcriptomics in the fly brain to compare Sod3 expression in neurons and glia (Extended Data Fig. 2c) (https://scope.aertslab.org/#/Davie_et_al_Cell_2018/Davie_et_al_Cell_2018%2FAerts_Fly_AdultBrain_Filtered_57k.loom/gene).

Research involving human participants, their data, or biological material

Policy information about studies with human participants or human data. See also policy information about sex, gender (identity/presentation), and sexual orientation and race, ethnicity and racism.

Reporting on sex and gender	not applicable
Reporting on race, ethnicity, or other socially relevant groupings	not applicable
Population characteristics	not applicable
Recruitment	not applicable
Ethics oversight	not applicable

Note that full information on the approval of the study protocol must also be provided in the manuscript.

Field-specific reporting

Please select the one below that is the best fit for your research. If you are not sure, read the appropriate sections before making your selection.

☒ Life sciences ☐ Behavioural & social sciences ☐ Ecological, evolutionary & environmental sciences

For a reference copy of the document with all sections, see nature.com/documents/nr-reporting-summary-flat.pdf

Life sciences study design

All studies must disclose on these points even when the disclosure is negative.

Sample size	Sample size choice was based on previous studies from our group such as Plaçais et al. Nat. Com. 2017 , de Tredern et al. Cell Rep., 2021, and Rabah et al. Nat. Metab. 2023for behavior and live imaging experiments.
Data exclusions	Memory assays where fewer than 6 flies were recovered after the behavioral test were excluded.
Replication	Each behavioral assay was replicated in at least 2 independent experiments. Imaging experiments were replicated in at least 4 independent experiments. All replications were successful.<br>All quantification were performed unblinded. Statistical parameters including the definitions and exact value of n (e.g. number of group of flies (behavior experiments), number of brains (in vivo live-imaging), deviations and p values are reported in the figures and corresponding figure legends. Statistical analysis was carried out using Prims8 (Graph Pad Software). Comparisons between two groups were performed by unpaired two-sided Student's t-test, with results given as the value tx of the t distribution, where x is the number of degrees of freedom. Comparisons among three groups were performed by one-way analysis of variance (ANOVA) with post hoc testing by the Newman-Keuls pairwise comparisons test between the experimental group and its controls (significance: $p<0.05$). ANOVA results are given as the value of the Fisher distribution F(x,y), where x is the number of degrees of freedom numerator and y is the total number of degrees of freedom denominator. Data are expressed as the mean ± s.e.m. with dots as individual values corresponding to a group of 40-50 flies analyzed together in a behavioral assay, to the response of a single recorded fly for calcium or H_2O_2 imaging.
Randomization	Flies were assigned to experimental groups based on genotypes.
Blinding	Data collection and analysis was not performed blind. Blinding is not applicable because the investigator who set up the experiment is the same person doing the analyses. However, each experiment was associated with proper controls, and sample were collected and analyzed under identical conditions.

Reporting for specific materials, systems and methods

We require information from authors about some types of materials, experimental systems and methods used in many studies. Here, indicate whether each material, system or method listed is relevant to your study. If you are not sure if a list item applies to your research, read the appropriate section before selecting a response.

Materials & experimental systems

n/a	Involved in the study
☐	☒ Antibodies
☒	☐ Eukaryotic cell lines
☒	☐ Palaeontology and archaeology
☐	☒ Animals and other organisms
☒	☐ Clinical data
☒	☐ Dual use research of concern
☒	☐ Plants

Methods

n/a	Involved in the study
☒	☐ ChIP-seq
☒	☐ Flow cytometry
☒	☐ MRI-based neuroimaging

Antibodies

Antibodies used

Primary antibodies:
- rat anti-HA, Roche, # ROAHAHA 11867423001, clone 3F10.
- mouse anti-HA, Biolegend # 901513, clone 16B12.
- mouse anti-tubulin, Sigma Aldrich # T16199, clone DM1A.
- mouse anti-GFP Developmental Studies Hybridoma Bank, # DSHB-GFP-12A6, clone 12A6
Secondary antibodies:
- goat anti-rat Alexa 488 Invitrogen # A11029
- HRP anti-mouse Promega # W4021
- goat anti-rat Alexa 594 Invitrogen # A1107

Validation

- rat anti-HA: doi:10.1242/bio.060335
- mouse anti-HA: doi.org/10.1038/s41467-021-20898-x
- mouse anti-alpha tubulin: doi: 10.1111/acel.13137
- mouse anti GFP: doi: 10.3390/biology10080729

Animals and other research organisms

Policy information about studies involving animals; ARRIVE guidelines recommended for reporting animal research, and Sex and Gender in Research

Laboratory animals

Drosophila melanogaster flies were raised on standard food medium containing yeast, cornmeal and agar, on a 12h:12h light-dark cycle at 18°C with 60% humidity. The Canton-Special (CS) strain was used as the wild-type strain. All lines were out-crossed for at least three generations to flies carrying a CS wild-type background. The study was performed on 0-3-day-old adult flies. For behavior experiments, both male and female flies were used. For imaging experiments, female flies were used.
List of Drosophila strains:
tub-GAL80ts; alrm-GAL4 (de Tredern et al. 2021)
tub-GAL80ts; c739-GAL4 (Turrel et al. 2018)
tub-GAL80ts, 13F02-LexA; VT30559-GAL4 (de Tredern et al. 2021)
tub-GAL80ts, 13F02-LexA; alrm-GAL4 (This study)
tub-GAL80ts; alrm-GAL4, VT30559-GAL4 (This study)
86E01-LexA (Bloomington Drosophila Stock Center (BDSC) 54287)
tub-GAL80ts, 86E01-LexA; VT30559-GAL4 (This study)
UAS-NoxRNAi HMS00429 (BDSC 32433)
UAS-NoxRNAi KK111991 (Vienna Drosophila Resource Center (VDRC) 102559)
UAS-G6PDRNAi KK108898 (VDRC 101507)
UAS-PgdRNAi HMC05959 (BDSC 65078)
UAS-PglsRNAi HMS02626 (BDSC 42933)
UAS-nAChRa7RNAi JF02570 (BDSC 27251)
UAS-nAChRa7RNAi KK108471 (VDRC 100756)
UAS-Sod3RNAi GD4801 (VDRC 37793)
UAS-Sod3RNAi GD3774 (VDRC 8760)
UAS-Glut1RNAi KK108683 (VDRC 101365)
UAS-AQPRNAi HMC03266 (BDSC 51504)
UAS-AQPRNAi KK102737 (VDRC 109314)
UAS-Prx2RNAi HMS00935 (BDSC 34971)
UAS-Prx2RNAi VSH330046 (VDRC 330046)
UAS-Trx2RNAi HMS00989 (BDSC 34019)
UAS-Trx2RNAi HMS00603 (BDSC 33721)
UAS-Trxr1RNAi HMS00784 (BDSC 32984)
UAS-Trxr1RNAi GD9738 (VDRC 47308)
UAS-ApplRNAi JF02878 (BDSC 28043)
UAS-ApplRNAi KK102543 (VDRC 108312)
UAS-Aß42 (Finelli et al. 2004)
UAS-GCaMP6f (BDSC 42747)
LexAop-GCaMP6f (BDSC 44277)
LexAop-GCaMP6f; UAS-Aß42 (This study)

UAS-GCaMP6f; UAS-nAChRa7RNAi JF02570 (This study)
LexAop-roGFP-Tsa2DCR (This study)
LexAop-roGFP-Tsa2DCR; UAS-Glut1RNAi KK108683 (This study)
LexAop-roGFP-Tsa2DCR; UAS-NoxRNAi HMS00429 (This study)
LexAop-roGFP-Tsa2DCR; UAS-G6PDRNAi KK108898 (This study)
LexAop-roGFP-Tsa2DCR; UAS-nAChRa7RNAi JF02570 (This study)
LexAop-roGFP-Tsa2DCR; UAS-Sod3RNAi GD4801 (This study)
LexAop-roGFP-Tsa2DCR; UAS-AQPRNAi HMC03266 (This study)
LexAop-roGFP-Tsa2DCR; UAS-ApplRNAi JF02878 (This study)
LexAop-roGFP-Tsa2DCR; UAS-Aß42 (This study)
APPL-HA (This study)
APPL-HA; tub-GAL80ts; VT30559-GAL4 (this study)
y ApplH(535)R,H(539)R (this study)
PBac{y[+]-attP-3B}VK00033 (BDSC 9750)
UAS-human-secreted-catalase (Dhawan et al. 2021)
UAS-BibRNAi GD1049 (VDRC 8893)
UAS-BibRNAi JF02771 (BDSC 27691)
UAS-DripRNAi GD1936 (VDRC 51936)
UAS-DripRNAi HMC0945 (BDSC 44661)
UAS-PripRNAi GD3499 (VDRC 8124)
UAS-PripRNAi HMC03097 (BDSC 50695)

Wild animals	The study did not involve wild animal
Reporting on sex	For behavior experiments, mixed populations of males and females were used. For imaging experiments, females were used.
Field-collected samples	The study did not involve samples collected from the field
Ethics oversight	No ethical approval or guidance was required since in this study we used Drosophila melanogaster.

Note that full information on the approval of the study protocol must also be provided in the manuscript.
