# Supplementary material for: Astrocyte-to-neuron H2O2 signalling supports long-term memory formation in Drosophila and is impaired in an Alzheimer’s disease model
Source: Nat Metab. 2025 Jan 24;7(2):321–35. doi: 10.1038/s42255-024-01189-3 (PMC11860231; doi:10.1038/s42255-024-01189-3)

APPL-HA

First column in Extended Fig. 7a

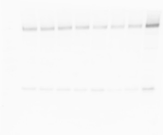

Tubulin
First column in Extended Fig. 7a

Molecular weight markers

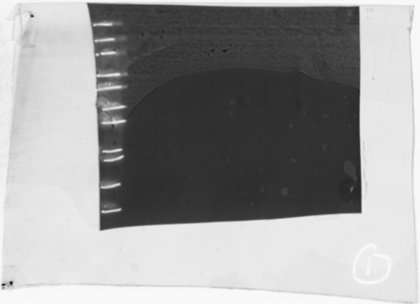

Supplement: Supplementary file 18 — Unprocessed gels for Extended Data Fig. 7. [file 42255_2024_1189_MOESM18_ESM.pdf]
